# More than Just a Game: A Longitudinal Pilot Study on the Outcome Effects of Home-Based Digital Cognitive Rehabilitation in Outpatients with Mild Cognitive Impairment

**DOI:** 10.3390/brainsci16060582

**Published:** 2026-05-29

**Authors:** Annalisa Magnani, Luca Bassi, Antonia Pierobon, Daniela Mancini, Valeria Torlaschi, Roberto Maestri, Pierluigi Chimento, Cira Fundarò, Marina Maffoni

**Affiliations:** 1 Department of Psychology, Catholic University of the Sacred Heart, 20123 Milan, Italy; annalisa.magnani@unicatt.it; 2 Department of Psychology, Sigmund Freud University, 20143 Milan, Italy; lucabbbassi@gmail.com; 3 Psychology Unit, Montescano Institute, Istituti Clinici Scientifici Maugeri IRCCS, 27040 Montescano, Italy; daniela.mancini@icsmaugeri.it (D.M.); valeria.torlaschi@icsmaugeri.it (V.T.); marina.maffoni@icsmaugeri.it (M.M.); 4 Department of Biomedical Engineering, Montescano Institute, Istituti Clinici Scientifici Maugeri IRCCS, 27040 Montescano, Italy; roberto.maestri@icsmaugeri.it; 5 Neuropsychophysiology Unit, Montescano Institute, Istituti Clinici Scientifici Maugeri IRCCS, 27040 Montescano, Italy; pierluigi.chimento@icsmaugeri.it (P.C.); cira.fundaro@icsmaugeri.it (C.F.)

**Keywords:** digital cognitive rehabilitation, home-based intervention, subjective cognitive decline, mild cognitive impairment, neuropsychological assessment, cognitive and psychological outcomes, reaction times

## Abstract

**Highlights:**

**What are the main findings?**

- Standardized neuropsychological and psychological outcomes remained overall stable over time, with no significant differences between the intervention and control groups.
- Training-derived performance metrics showed variable, participant-specific patterns, with improvements in task speed observed in some cases.

**What are the implications of the main findings?**

- Training-derived metrics may offer complementary information on individual performance changes during digital cognitive rehabilitation.
- Individualized home-based digital cognitive interventions appear feasible in people with MCI, but further investigation in larger and more controlled studies is warranted.

**Abstract:**

**Background**: This pilot study investigated the potential effects of an individualized home-based cognitive training program delivered through the Neurotablet^®^ in individuals with Mild Cognitive Impairment (MCI). **Methods:** Fourteen outpatients were assigned to the experimental group (n = 7) or to the waiting-list control group (n = 7). Standardized neuropsychological and psychological measures were collected at baseline and follow-up. Outpatients of the experimental group completed an eight-week personalized home-based digital rehabilitation protocol targeting multiple cognitive domains. Longitudinal changes in standardized outcomes were analyzed using generalized linear mixed models. In addition, individual MCI trajectories were examined qualitatively by comparing MCI subtype between baseline and follow-up, whereas training performance was analyzed using within-participant linear regression models. **Results:** Standardized neuropsychological outcomes remained overall stable over time, with no significant differences between groups. By contrast, analyses of training performance revealed heterogeneous patterns across participants, with significant session-related changes emerging in some individuals. In particular, some tasks showed reductions in reaction times across sessions, suggesting changes in task efficiency with repeated practice. These findings highlight the heterogeneity of cognitive trajectories in MCI and suggest that individualized digital training may be associated with measurable changes in task performance, even in the absence of detectable changes in standardized neuropsychological measures. **Conclusions:** Despite the presence of limitations, digital cognitive interventions may offer a feasible approach to support the delivery of individualized cognitive rehabilitation in individuals reporting cognitive complaints. Further studies with larger samples, more detailed monitoring of training variables and engagement are needed to better clarify the potential cognitive impact of such interventions.

## 1. Introduction

Neurocognitive disorders (NCDs) are becoming increasingly relevant in the context of demographic changes and improved survival following acute and chronic diseases, drawing attention toward early phases of cognitive decline and subtle cognitive difficulties [1,2,3,4,5]. Rather than being conceptualized solely through distinct diagnostic categories, cognitive decline is increasingly considered as unfolding along a continuum ranging from subjective complaints to mild and overt impairment, characterized by heterogeneous clinical expressions and variable impact on everyday functioning [6]. Accordingly, clinical practice and research have progressively shifted toward the earliest phases of cognitive decline, aiming to better understand the factors that modulate its evolution and to identify potential windows for intervention before stable functional impairments emerge [4,5,7].

Within this scenario, Subjective Cognitive Decline (SCD) has gained relevance. SCD is defined as the subjective perception of a decline in cognitive abilities in the absence of objective deficits detectable through standard neuropsychological testing [8,9,10]. Nevertheless, neuropsychological testing often reveals minor but persistent cognitive deficits that approach or meet the criteria for Mild Cognitive Impairment (MCI) [11,12]. MCI—affecting approximately 19–24% of the geriatric population [13,14]—is characterized by objective decline in one or more cognitive domains alongside substantially preserved independence in daily activities [11,12], and can be further classified as amnestic or non-amnestic and as single-domain or multidomain—with implications for prognosis and intervention planning [11]. This transitional condition is a primary target for secondary prevention and early rehabilitative intervention [4,8,9]. Importantly, this continuum should be carefully interpreted as it can be modulated by interacting factors including cognitive reserve, medical comorbidities, lifestyle, and psychological health [3,4,8,15,16]. In particular, depressive and anxiety symptoms may amplify the perception of cognitive difficulties, making their assessment an essential component of the clinical framework [7,17,18]. This heterogeneity complicates early identification, risk stratification, and intervention selection, highlighting the need for an integrated framework that jointly considers cognitive and psychological dimensions [7,19].

From a clinical and research perspective, cognitive rehabilitation has traditionally been directed primarily toward individuals with objectively measurable impairment, such as those with MCI, where deficits can be quantified through standardized neuropsychological testing and monitored over time [1]. However, even within this clinical population, the literature highlights an ongoing debate regarding the optimal structure of cognitive training, namely whether interventions should focus on a single cognitive domain or adopt a broader multidomain approach [20,21]. Meta-analytic data indicate that both formats may be associated with benefits, although results remain heterogeneous and appear to depend on baseline characteristics and intervention features [20,21,22,23,24]. In this context, multidomain training represents a clinically meaningful and pragmatic choice, more consistent with the multifaceted nature of everyday cognitive functioning and particularly appropriate to the heterogeneous profile of individuals with early MCI [7,11,25].

Alongside the consolidation of evidence for cognitive training in MCI, there has been a growing emphasis on the delivery format of rehabilitative interventions. Digital platforms for cognitive rehabilitation—including computerized programs, tablet-based applications, and virtual reality systems—have gained increasing relevance in recent years, offering the possibility of delivering structured, adaptive, and remotely monitored training programs in home-based settings [23,26,27]. Compared to traditional in-person rehabilitation, digital or computerized cognitive training programs, especially when conducted in a home-based setting, offer several advantages [20]. These include increased accessibility for people with mobility impairments or who reside in underserved areas, greater scheduling flexibility, and the possibility of higher training frequency and ecological integration into the patient’s daily routine [20,28,29,30]. Furthermore, modern platforms can incorporate self-adaptive algorithms that dynamically adjust task difficulty based on real-time performance, thus supporting individualized training pathways [29,30]. Despite these benefits, the home-based setting introduces specific challenges, such as limited direct supervision, variable adherence, and reduced control over environmental conditions during task performance—factors that must be carefully considered when interpreting intervention outcomes [26,30].

A related and clinically important issue concerns the sensitivity of outcome measures used to assess the effectiveness of cognitive training in MCI populations. As noted, the heterogeneity of MCI subtypes entails distinct impairment patterns, rates of progression, and possible treatment responsiveness [11]. Inter-individual variability may mask or weaken intervention effects present in particular subgroups when individuals with such heterogeneous profiles are grouped together without prior stratification, resulting in non-significant aggregate outcomes even when significant changes occur at the individual level [31,32,33]. These considerations have prompted interest in complementary sources of outcome data, including performance metrics derived from the training tasks themselves. Reaction times, error rates, and task completion times recorded across training sessions can offer a precise, ecologically proximal window into individual cognitive trajectories, capturing within-person changes in task efficiency that may remain undetected by conventional assessment instruments [23,26,31].

On the basis of the aforementioned considerations, the primary aim of this study is to evaluate the feasibility and effectiveness of a home-based digital cognitive rehabilitation program in outpatients classified as having MCI, by comparing changes in psychological and neuropsychological test performance between participants receiving the intervention and those allocated to a waiting-list control group (between-group comparisons). The secondary objectives are as follows:

1. To describe the sociodemographic and clinical features of the overall sample and of each study group.
2. To evaluate within-group changes over time in psychological and neuropsychological outcomes in both the intervention and waiting-list groups.
3. To examine, within the intervention group, individual-level longitudinal trends in neuropsychological outcomes, rehabilitation exercise performance, and adherence to the home-based digital cognitive rehabilitation protocol.

Our hypothesis is that a structured digital cognitive rehabilitation program is beneficial for this clinical population and that training-derived metrics can provide objective, quantitative evidence of improvements in targeted cognitive functions, thereby supporting a more operational and measurable approach to early cognitive rehabilitation.

## 2. Materials and Methods

### 2.1. Study Design

The present study is embedded within the broader MASCoD project (Multidimensional Assessment of Subjective Cognitive Decline), an ongoing clinical–research program aimed at improving the early identification, characterization, and clinical management of individuals presenting with subjective cognitive complaints who may be at increased risk of future cognitive decline. The primary aim of the MASCoD project is to provide a structured, multidimensional screening approach that supports clinicians in differentiating subjective complaints suggestive of prodromal neurocognitive disorders from those related to other conditions, guiding personalized diagnostic and rehabilitative pathways. The full study protocol has been registered on ClinicalTrials.gov (ID: NCT05815329) [34], and the screening tool with its preliminary validation has been described elsewhere [7,35]. Within this wider framework, the current study adopts a prospective, two-arm, open label randomized controlled design to conduct a pilot investigation to evaluate the effects of a digitally delivered cognitive intervention in individuals showing objective cognitive vulnerabilities at baseline.

### 2.2. Ethical Considerations

The study was approved by the Ethics Committee of the Istituti Clinici Scientifici (ICS) Maugeri IRCCS (Protocol CE 2666, 26 July 2022) and was conducted in accordance with principles of transparency and scientific rigor, as well as the ethical standards outlined in the Declaration of Helsinki [36]. Participation was voluntary and subject to the signature of an informed consent form and consent to data processing. Additionally, participants were informed of their right to withdraw from the study at any time without consequences. No financial compensation was provided.

### 2.3. Participants and Procedure

Participants were consecutively recruited from the Centers for Cognitive Disorders and Dementia (CCDDs) of the IRCCS ICS Maugeri (Montescano Institute, Pavia, Italy), among outpatients who required a neurological consultation due to self-reported cognitive difficulties affecting their daily functioning [37]. In accordance with institutional Diagnostic–Therapeutic Care Pathways (DTCPs)—which are aligned with international guidelines for the management of cognitive disorders [38]—all patients underwent a standardized neurological evaluation. During the clinical interview, the neurologist verified eligibility criteria defined within the MASCoD project framework. Specifically, inclusion criteria were: (i) individuals aged 55 years or older, (ii) Italian educational background, (iii) reported subjective cognitive complaints in the absence of a previously diagnosed neurological or cognitive disorder, (iv) acceptance of the study objectives and procedures. Exclusion criteria were: (i) presence of severe medical conditions (e.g., severe cardiovascular, respiratory, or neoplastic diseases), (ii) a current or past major psychiatric disorder according to DSM-5-TR [12], (iii) a prior diagnosis of cognitive impairment, (iv) sensory deficits (visual, perceptual, or auditory) that could interfere with the assessment, (v) illiteracy.

After providing written informed consent, participants enrolled on the basis of a clinical suspicion of SCD underwent a multidimensional baseline assessment (T_0_), including administration of the MASCoD screening schedule, an extensive neuropsychological evaluation, and brain ^18^F-FDG PET imaging—the latter performed when objective cognitive vulnerabilities emerged from the assessment, defined as Equivalent Scores of 0 or 1 at least in two test scores across cognitive domains [7,35]. Neuropsychological testing was conducted to objectively characterize cognitive functioning across multiple domains using standardized and validated tools consistent with national and international recommendations [38,39].

Although several participants demonstrated cognitive performance within normal limits, consistent with SCD, a subset exhibited subtle yet consistent weaknesses, with scores approaching diagnostic criteria for MCI [12,13]. This pattern—characterized by subjective cognitive complaints accompanied by emerging objective vulnerabilities—represents a clinically relevant condition situated along the AD continuum and is considered of particular interest for secondary prevention strategies [8,9]. To ensure clinical and ethical appropriateness [7,40,41], only participants presenting objective cognitive vulnerabilities at baseline were considered eligible for the cognitive rehabilitation intervention and were therefore included in the present randomized study. Individuals with preserved cognitive performance received usual care and clinical monitoring according to standard practice and contributed exclusively to the primary observational aims of the MASCoD project [7,35].

MCI subtypes were classified according to the criteria proposed by Petersen [12] and the International Working Group on Mild Cognitive Impairment [13]. Participants were categorized as amnestic MCI (a-MCI) when impairment primarily involved episodic memory, operationalized through the long-term verbal memory measures described in Section 2.5, and as non-amnestic MCI (na-MCI) when memory was relatively preserved and impairment involved other cognitive domains. Each subtype was further subclassified as single-domain when impairment was confined to the index domain, or multidomain when one or more additional cognitive domains were also impaired according to the same normative criteria.

**Figure 1:**
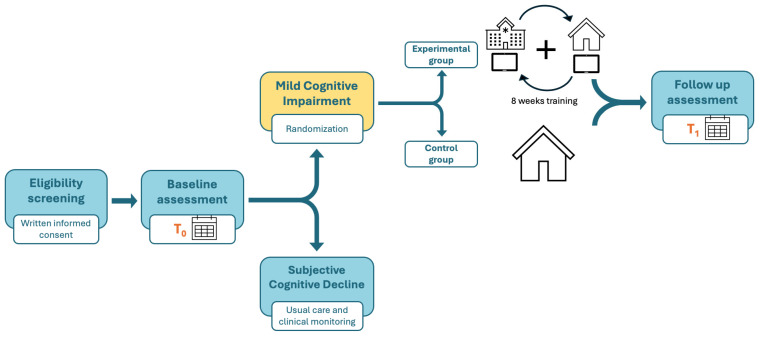
Schematic representation of participant flow through the study. After eligibility screening, informed consent, and baseline assessment (T_0_), participants were classified according to cognitive status. Individuals presenting objective cognitive vulnerabilities consistent with Mild Cognitive Impairment were randomized to the experimental or waiting-list control group, whereas participants with Subjective Cognitive Decline received usual care and clinical monitoring. Follow-up assessment (T_1_) was conducted at six months.

Enrolled participants eligible for rehabilitation were then randomly allocated in a 1:1 ratio to either (i) an experimental group (EG), undergoing a home-based digital cognitive rehabilitation program, or (ii) a waiting-list control group (CG). Randomization was centrally managed by the Bioengineering Department using a dedicated computer-generated randomization sequence. The allocation sequence was concealed until assignment, and the enrolling investigators were informed of each participant’s group allocation only at the beginning of the training phase. Participants allocated to the experimental group received an eight-week home-based digital cognitive rehabilitation intervention delivered using the Neurotablet^®^ (Neurab s.r.l., Trentino-Alto Adige, Italy). During the eight-week intervention period, participants performed cognitive training exercises independently at home for approximately 30 min per day, five days per week. In parallel, participants attended a weekly face-to-face session with a psychologist, lasting approximately 60 min, aimed at monitoring progress, identifying difficulties, and supporting the development of compensatory strategies applicable to daily life. A follow-up evaluation (T_1_) was conducted at six months. Figure 1 summarizes the overall study workflow.

### 2.4. Intervention Tool and Rehabilitation Outcomes

The digital cognitive rehabilitation intervention was delivered using the Neurotablet^®^, a device specifically developed to support cognitive rehabilitation in individuals with congenital or acquired neurological conditions—both in clinical settings and in home-based contexts. The system is designed to assist clinicians in planning individualized rehabilitation programs, delivering structured cognitive training, and monitoring patient performance over time. Its clinical effectiveness has been previously documented in rehabilitative settings [29,30].

In brief, the Neurotablet^®^ is a multi-platform system optimized for neurorehabilitation and consists of a tablet equipped with integrated connectivity features enabling secure data transmission, remote monitoring, and program customization. The platform includes a library of over 40 modular cognitive exercises targeting multiple cognitive domains, including attention, memory, perception, executive functions, language, and unilateral spatial neglect. By manipulating several task parameters—such as the number and characteristics of stimuli, reaction time constraints, and trial length—the system can generate more than 10,000 levels of difficulty, thereby enabling personalized training intensity. Personalization and treatment adherence are supported by two key features. First, the Neurotablet^®^ incorporates a self-adaptive algorithm that dynamically adjusts task difficulty in real time based on user performance. Second, the system provides real-time feedback during task execution and records detailed performance metrics (e.g., error rates, reaction times, task completion times).

The exercises of each individual rehabilitation pathway were defined based on each participant’s cognitive profile emerging from the baseline neuropsychological assessment, with particular emphasis on identified areas of difficulty [28,42,43]. For the purposes of the present study, a subset of exercises commonly shared across participants was selected, covering most cognitive domains addressed by the intervention, while preserving the individualized and adaptive nature of the rehabilitation program. For each exercise, the Neurotablet^®^ platform automatically recorded detailed performance metrics during the execution of home-based training sessions. These metrics included reaction times, task completion times, total training time per session, and exercise difficulty levels, which were collected at each training session and used to characterize longitudinal performance trends and adherence to the protocol during rehabilitation. The selected exercises are described below:

- Multiple alert—Participants are shown geometric shapes (e.g., triangles, squares, diamonds, circles) in various colors. They are instructed to tap the screen as quickly as possible whenever a predefined target stimulus appears, while ignoring all non-target figures.
- Stroop color—This task is based on the classic Stroop paradigm. At lower difficulty levels, participants see color words and must press the button corresponding to the meaning of the word. At higher levels, the task requires inhibitory control: participants must respond according to the ink color of the word, disregarding its meaning.
- Flow free—Participants are shown a grid with pairs of dots of the same color. They must connect each pair by drawing lines that do not overlap or cross, ensuring that the entire grid is filled without leaving empty spaces.
- Go/no-go divided screen—Across a series of trials, participants are shown a screen divided either horizontally or vertically. A stimulus (a ball) appears in one of the two sections, and participants must touch the opposite, empty section as quickly as possible. In some trials, an auditory distractor (a cat meowing) is presented, signaling that participants should inhibit their response in the subsequent trial.
- Visual pathway memory—Participants are shown an animated sequence in which a path is drawn by connecting a series of points on a grid. When the animation disappears, they must reproduce the same path by tracing it with their finger in the correct order. Task difficulty increases progressively by enlarging the grid and increasing the number of points and connections.
- Tangram—Participants are asked to reconstruct a target figure displayed on the screen using a set of geometric pieces.

### 2.5. Cognitive and Psychological Outcome Measures

After the collection of sociodemographic (age, gender, education, marital status, living condition) and clinical information (body mass index, comorbidities, lifestyle and risk factors), all participants underwent a multidimensional baseline assessment. The same assessment battery was re-administered at the 6-month follow-up.

Cognitive functioning was assessed through a comprehensive neuropsychological battery. Global cognitive functioning was evaluated using the Mini-Mental State Examination (MMSE) [44] and the Addenbrooke’s Cognitive Examination-III (ACE-III) [45]. Executive functions and attentional control were assessed with the Frontal Assessment Battery (FAB) [46], the Trail Making Test (TMT-A and TMT-B) [47], and the Stroop Color–Word Test [48]. Short-term and working memory, both verbal and visuo-spatial, were examined using the Digit Span Forward and Backward (DSF and DSB) and the Corsi Block-Tapping Test Forward and Backward (CSF and CSB) [49]. Learning and long-term memory, both verbal and visuo-spatial, were assessed using Rey’s Auditory Verbal Learning Test (RAVLT immediate and recall) [50], and the recall trial of the Rey–Osterrieth Complex Figure (ROCF delayed recall) [51]. Language abilities were evaluated through phonemic and semantic verbal fluency tasks [52]. Visuospatial abilities and constructive praxis were evaluated using the copy trial of the Rey–Osterrieth Complex Figure (ROCF copy) [51] and the Clock Drawing Test (CDT) [53].

Psychological functioning was assessed using validated self-report questionnaires. Depressive symptoms were measured with the Patient Health Questionnaire-9 (PHQ-9) [54,55], anxiety symptoms with the Generalized Anxiety Disorder-7 (GAD-7) [56,57]. In addition, subjective perception of cognitive changes was assessed with the Cognitive Function Instrument (CFI, self-report) [58] and with the Multidimensional Assessment of Subjective Cognitive Decline (MASCoD) [7,35].

A comprehensive description of all assessed cognitive and psychological domains, including the instruments used, their administration procedures, and scoring methods, is provided in the Supplementary Materials File S1.

### 2.6. Data Analysis

All statistical analyses were performed using SAS/STAT software, version 9.4 (SAS Institute Inc., Cary, NC, USA). Radar charts for the qualitative visualization of individual longitudinal neuropsychological profiles were created using Microsoft Excel version 16.108.2 (Microsoft Corporation, Redmond, WA, USA).

The normality of continuous variables was assessed using the Shapiro–Wilk test. As several variables deviated from normality and given the pilot nature of the study with a small sample size, continuous variables were reported as median and interquartile range (Q1, Q3), while categorical variables were presented as counts (%). Missing data were not imputed. Between-group differences at baseline were evaluated using chi-squared or Fisher’s exact tests for categorical variables and Mann–Whitney U tests for continuous variables. For hypothesis testing on neuropsychological outcomes, raw scores were corrected for age, sex, and years of education, when appropriate, according to published Italian normative data, and corrected scores were used in all analyses. Effect sizes for within-group changes were assessed using the median of paired differences as a descriptive measure of change and the rank-biserial correlation (RBC) as a nonparametric measure of effect size. RBC values range from −1 to +1, with values closer to 0 indicating smaller effects and values closer to −1 or +1 indicating larger effects. To evaluate the effect of treatment over time, a generalized linear mixed model (GLMM) was fitted to account for the longitudinal design and within-subject correlations. The model included Group (EG vs. CG), Time (T_0_ and T_1_), and their interaction (Group × Time) as fixed effects. Results from the GLMM are reported as model-based estimated means and 95% confidence intervals, back-transformed to the original scale, together with the corresponding *p* values for Time, Group, and the Group × Time interaction with F statistics, and partial eta squared as an effect size measure. All statistical tests were two-tailed, and significance was set at *p* < 0.05.

In addition to group-level analyses, individual longitudinal neuropsychological profiles were examined through a qualitative observational framework. Specifically, each participant’s MCI subtype was independently classified at baseline (T_0_) and follow-up (T_1_) using the same Petersen and Winblad criteria, enabling a direct comparison between the two timepoints and the explicit identification of changes in the specific cognitive functions involved. To complement this categorical reclassification, individual radar plots were generated. Besides standardized clinical outcomes, training-derived performance metrics were analyzed. For each participant and task, reaction times were analyzed at the individual level using linear regression models including task difficulty and session number as predictors. To reduce trial-level noise and the influence of local outliers, reaction times were preprocessed using spline-based smoothing procedures. Adherence to the home-based digital cognitive rehabilitation program was evaluated by calculating the percentage of completed training time relative to the expected training duration (30 min of training per day, 5 days per week, for 8 consecutive weeks, corresponding to an expected total of 1200 min of training).

## 3. Results

### 3.1. Sociodemographic and Clinical Characteristics of the Sample

As shown in Table 1, participants in the EG were significantly older than those in the CG (median age 77.0 vs. 62.0 years, *p* = 0.011). Marital status did not differ significantly between groups (*p* = 0.07), although the EG included a higher proportion of widowed participants (57.1% vs. 0%), whereas all CG participants were married or cohabiting (100%). Similarly, employment status did not differ significantly between groups (*p* = 0.19), although all EG participants were retired, while 42.9% of the CG participants were employed. The main source of social and family support also did not differ significantly (*p* = 0.10), though EG participants more frequently relied on a son or daughter (71.4% vs. 14.3%), and CG participants more often reported a spouse or partner as their primary source of support (85.7% vs. 28.6%). Beyond these socio-familial aspects, the two groups appeared well balanced: no significant differences were observed between groups with respect to years of schooling, anthropometric measures (weight, height, BMI), gender distribution, smoking status, physical activity, overweight condition, alcohol consumption, family history of non-communicable diseases, or major comorbidities including diabetes, hypertension, and dyslipidemia (all *p* > 0.05). Overall, aside from age, the two groups were comparable in terms of clinical, anthropometric, and lifestyle characteristics at baseline.

### 3.2. Adherence to the Intervention

During the intervention period, no dropouts were observed, and all EG participants completed the scheduled in-person sessions. Regarding the home-based digital training, six of seven participants completed approximately 70% or more of the expected training time. Adherence showed substantial inter-individual variability, with completed training ranging from 35.8% to 214.1% of the expected dose. Individual adherence values are reported in Table 2.

### 3.3. Neuropsychological and Psychological Outcomes

Descriptive statistics for baseline (T_0_) and follow-up (T_1_) scores, within-group changes, and rank-biserial correlations are reported in Table 3a for completeness. Inferential results are based on the GLMM and are reported in Table 3b as estimated marginal means (EMM) with the main effects of Group, Time, and the Group × Time interaction.

The GLMM revealed a significant main effect of Group only for the ACE-III (F(1,12) = 5.26, *p* = 0.047, η^2^p = 0.30), with the EG scoring higher than the CG across both time points. However, this difference was already present at baseline and does not reflect an intervention effect. No significant main effects of Time were observed for any outcome (all *p* > 0.05). The Group × Time interaction did not reach statistical significance for any cognitive or psychological measure (all *p* > 0.05). Nevertheless, TMT-A (F(1,12) = 3.69, *p* = 0.079, η^2^p = 0.24) and TMT-B (F(1,12) = 4.27, *p* = 0.066, η^2^p = 0.26) showed trends toward significance with large effect sizes. For both measures, the EMMs indicated a reduction in completion times from T_0_ to T_1_ in the EG and a parallel increase in the CG, suggesting a trend toward differential improvement in processing speed and cognitive flexibility favoring the experimental intervention.

### 3.4. Neurotablet^®^ Training

Given the individualized and adaptive nature of the Neurotablet^®^ training, aggregated group-level analyses were not conducted. Instead, longitudinal performance trends were examined at the individual level using linear regression models. For each participant and task, reaction times were modeled as a function of session number and task difficulty. The Session coefficient (β_2_) was interpreted as an index of performance change across training sessions, with negative β_2_ values indicating progressive reduction in reaction times over time. Only β_2_ estimates with *p* < 0.05 were considered statistically significant. For Multiple alert (n = 4), significant Session effects were observed in 4 out of 4 participants, and all significant β_2_ coefficients were negative. For Stroop color (n = 3), significant Session effects were observed in 1 out of 3 participants, with a positive β_2_ coefficient. For Flow free (n = 5), significant Session effects were observed in 4 out of 5 participants, and all significant β_2_ coefficients were negative. For Go/no-go divided screen (n = 4), significant Session effects were observed in 1 out of 4 participants, with a negative β_2_ coefficient. For Visual pathway memory (n = 6), significant Session effects were observed in 2 out of 6 participants: one participant showed a positive β_2_ coefficient and one participant showed a negative β_2_ coefficient. For Tangram (n = 6), significant Session effects were observed in 2 out of 6 participants, and both significant β_2_ coefficients were negative.

**Figure 2:**
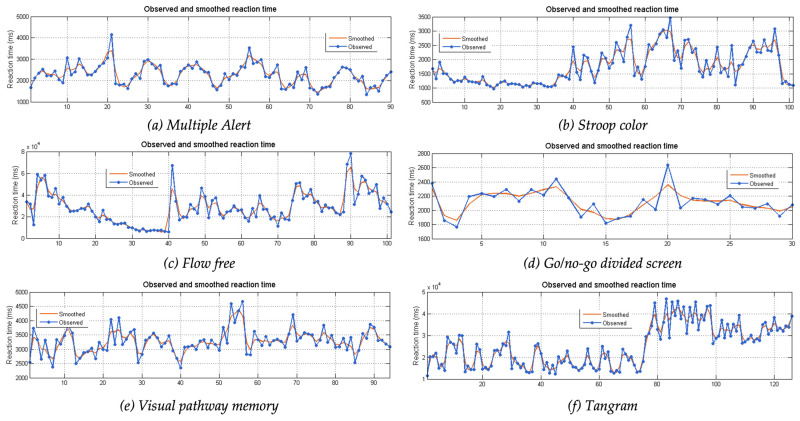
Observed and smoothed reaction times (ms) across sessions and exercises (**a**–**f**) for participant 1. Blue markers represent observed reaction times, while the orange line represents the smoothed trend. For each task, reaction times were modeled as a function of task difficulty and session number. The Session coefficient (β_2_) represents the change in reaction time per session after controlling for difficulty. Significant negative β_2_ indicates progressive improvement in task efficiency despite increasing difficulty. Positive β_2_ reflects the steep adaptive escalation of task demands rather than declining performance. (**a**) Multiple alert: β_2_ = −5.61, df = 87, F-statistic = 24.1, *p* < 0.0001, Adjusted R-Squared = 0.342; (**b**) Stroop color: β_2_ = 6.2971, df = 98, F-statistic = 55.3, *p* < 0.0001, Adjusted R-Squared = 0.521; (**c**) Flow free: β_2_ = −179.33, df = 98, F-statistic= 17.0, *p* < 0.0001, Adjusted R-Squared = 0.242; (**d**) Go/no-go divided screen: β_2_ = −1.8387, df = 27, F-statistic = 0.391, *p* = 0.68, Adjusted R-Squared = 0.0282; (**e**) Visual pathway memory β_2_ = 1.3768, df = 91, F-statistic = 6.52, *p* = 0.0022, Adjusted R-Squared = 0.106; (**f**) Tangram β_2_ = −42.203, df = 123, F-statistic = 178, *p* < 0.0001 Adjusted R-Squared = 0.739.

Figure 2 shows data from participant 1, who emerged as the most informative case. Complete regression outputs, including all coefficients and model fit indices for each participant and task, are reported in the Supplementary Materials File S2.

### 3.5. Individual Longitudinal Cognitive Trajectories

Individual longitudinal profiles are summarized in Table 4, which reports for each participant the MCI subtype at T_0_ and T_1_, the specific cognitive functions impaired at each timepoint, and the qualitative trajectory category. Corresponding radar plots, allowing visual inspection of the cognitive profile changes across the two timepoints, are reported in the Supplementary Materials File S3.

Across the Experimental Group (n = 7), trajectories appeared heterogeneous but mostly oriented toward a reduction in impaired cognitive functions. Three participants (EG1, EG4, EG5) showed a reduction in deficits within the same MCI subtype, one participant (EG6) showed an analogous pattern in combination with a shift toward a milder MCI subtype, and one participant (EG2) no longer met formal MCI criteria at follow-up. Two participants (EG3, EG7) presented a within-subtype progression of impaired functions.

Across the Control Group (n = 7), trajectories also appeared heterogeneous, with a comparatively higher proportion of stable-to-worsening patterns. Two participants (CG3, CG4) showed a within-subtype reduction in deficits, and one participant (CG2) showed a profile reorganization without net change in the number of impaired functions. Two participants (CG5, CG7) presented a within-subtype progression of deficits, and two participants (CG1, CG6) showed an MCI shift accompanied by widespread cognitive decline.

## 4. Discussion

### 4.1. Discussion of the Main Findings

The present study investigated the potential effects of an individualized home-based cognitive training program delivered through the Neurotablet^®^ platform in individuals diagnosed with MCI. Overall, the findings reveal a complex picture: while standardized neuropsychological and psychological measures remained stable over time, training-derived performance metrics showed heterogeneous patterns across participants, with session-related changes emerging in some individuals. This heterogeneity is consistent with recent literature emphasizing the multifaceted nature of cognitive trajectories in early cognitive decline [9,10,19] and reflects both the individualized structure of the intervention and the clinical variability inherent to MCI as a transitional condition.

Before interpreting these outcomes, it is important to consider adherence to the training, as it provides essential context for evaluating the feasibility and potential impact of the intervention. In the present study, the absence of dropouts and the completion of all scheduled in-person sessions support the feasibility of the intervention. With respect to home-based training adherence, six of the seven EG participants completed approximately 70% or more of the expected home-based training time, supporting generally acceptable adherence. Only one participant completed less (35.8%). Despite this overall acceptable adherence, completion varied markedly across participants. This variability is consistent with previous findings from cognitive training and digital rehabilitation studies [59,60], and is particularly relevant in light of the sociodemographic differences observed between groups at baseline. Indeed, despite randomization, the small sample size likely produced this imbalance—a well-documented limitation of reduced-sample randomized design [61] which may have influenced both adherence to the intervention and the pattern of results. As reported in the Results section, EG participants were older, more frequently widowed, and more likely to rely on adult children as their primary support, whereas CG were generally younger, more often partnered, and more likely to report a cohabitating partner as their main source of support. Age is a particularly relevant factor here, both because older age is associated with greater vulnerability to cognitive decline and reduced neuroplastic capacity [62]—representing the most likely source of residual confounding in between-group comparisons given observed imbalance [63]—and because older participants typically face greater barriers to the use of digital platforms, including lower digital literacy, reduced experience with technology, lower perceived confidence, and greater dependence on external support [59,64]. Though Neurotablet^®^ was preconfigured to minimize technical demands, sustained home-based use still required basic interaction with the device and consistent engagement over time, and limited confidence with digital tools may therefore have affected not only adherence but also the quality and continuity of training exposure [20,65]. Therefore, social support represents a further relevant factor. Widowhood and social isolation are risk factors for both depressive symptoms and cognitive deterioration [66], and the type of support available in daily life may be especially important in home-based interventions [20,67]. Evidence from computerized cognitive training suggests that being partnered or cohabiting is associated with a greater likelihood of initiating and sustaining training [60]. In this sense, a cohabiting partner may provide daily reminders and encouragement, whereas adult children may offer more intermittent support, with potential consequences for engagement and adherence [3,4,17,68]. Lastly, in addition to the completion of a prescribed training dose, the recent literature highlights how adherence in digital rehabilitation programs is closely linked to training intensity and broader engagement-related constructs [69,70]. Usability, perceived usefulness, satisfaction and perceived cognitive effort when using the platform are all factors that should be taken into consideration [71], given that an adequate dose alone does not necessarily translate into cognitive benefit [20]. Therefore, the present results support the feasibility and acceptability of the intervention in terms of session completion, while individual differences in age, social support, digital confidence, and engagement quality may have additionally contributed to shaping adherence and treatment responsiveness.

Instead, at the group level, the GLMM did not show significant main effects or interactions for the standardized neuropsychological and psychological outcomes, suggesting overall stability across the observation period. Nonetheless, while no interaction was statistically significant, TMT-A and TMT-B showed trends toward significance with large effect sizes. Estimated marginal means for all measures showed a decrease in completion times from baseline to follow-up in the EG and a concurrent increase in the CG, indicating a potential differential trajectory favoring the experimental intervention in cognitive flexibility and processing speed. However, given the small sample size and the exploratory nature of these analyses, these findings are only preliminary signals that may provide useful indicators for future implementation. Globally, these results are consistent with the characteristics of individuals in early phases of cognitive decline, in which initial cognitive complaints may coexist with relatively preserved overall neuropsychological profile [9,10,18]. Moreover, as described in the introduction, MCI encompasses distinct clinical profiles—amnestic and non-amnestic, single-domain and multidomain [12]—and this clinical heterogeneity may have attenuated intervention effects at the group level [31,33]. The qualitative analysis of individual longitudinal trajectories may provide additional context for interpreting these group-level findings. In the EG, trajectories were heterogeneous but mostly oriented toward a reduction in impaired cognitive functions. Conversely, although some CG participants also showed reduced deficits or profile reorganization, the control group displayed a comparatively higher proportion of stable-to-worsening trajectories. Although these qualitative observations cannot provide evidence of training efficacy, they support the possibility that aggregate analyses may mask clinically relevant individual-level changes, particularly in small and heterogeneous samples [63].

Concurrently, the psychological measures remained substantially stable over time in both groups. This finding has different clinical implications for each group. For the EG, this stability may indicate that sustained adherence to cognitively demanding digital tasks did not produce adverse psychological effects—an important consideration given that repeated confrontation with one’s own cognitive limitations during training could increase frustration, performance anxiety, or heightened awareness of difficulties [72]. For the CG, this stability over the waiting-list period may suggest that the absence of active intervention did not lead to worsening of affective symptoms or increased subjective cognitive complaints. This is a noteworthy finding, as it suggests that being assigned to a non-treatment condition and waiting six months without targeted support did not foster feelings of neglect, heightened uncertainty about one’s cognitive status, or anticipatory anxiety regarding future decline [73,74].

In parallel, analyses of the training tasks revealed that, in some individuals, reductions in reaction times across sessions were observed, indicating changes in task performance during the training period. These findings suggest that repeated engagement with the training tasks may be associated with measurable changes in task efficiency. Specifically, session-related improvements were most consistently observed in tasks involving sustained and selective attention (Multiple alert) and visuospatial planning (Flow free, Tangram), whereas effects were less pronounced or absent in tasks requiring inhibitory control (Go/no-go divided screen) or complex memory processes (Visual pathway memory). This domain-specific pattern is noteworthy and could reflect the differential sensitivity of cognitive domains to training-related practice effects [22,23,32]. More generally, the observed reductions in reaction times may reflect increased efficiency in task processing, task-specific learning, or growing familiarity with the training paradigm across repeated sessions, as described in previous cognitive training studies [22,23], and supported by more recent evidence showing task-specific improvements without consistent transfer to standardized neuropsychological measures [32]. At the same time, the variability observed across participants likely reflects the clinical heterogeneity of the MCI group discussed above, which—encompassing different subtypes and impairment profiles—may differentially influence responsiveness to cognitive training and its outcomes [12,33,75].

A further point emerging from the present study concerns the relationship between performance changes in the training tasks and standardized neuropsychological outcomes. While some session-related trends were observed within the Neurotablet^®^ tasks, the neuropsychological measures remained overall stable over time. This discrepancy is consistent with findings from cognitive training research, where improvements in trained tasks do not necessarily translate into measurable gains on standardized neuropsychological tests, particularly in individuals in early or transitional phases of cognitive decline [22,23,26], and aligns with more recent evidence suggesting that digital cognitive tasks may capture subtle changes not detected by traditional neuropsychological assessments [31]. Likewise, this gap highlights important considerations about the appropriate outcome framework for evaluating cognitive rehabilitation in early MCI. While standardized neuropsychological tests are essential for clinical classification and diagnosis [1,28], they may not be the most sensitive indices of treatment response in populations with subtle or heterogeneous cognitive changes. Training-derived performance metrics—such as reaction times, error rates, and difficulty progression—could provide a complementary, task-proximal window into individual cognitive trajectories that may be more sensitive to intervention-related changes than standardized neuropsychological measures [29,30].

These considerations place the current work within a rapidly developing field characterized by growing interest in remotely delivered, personalized cognitive interventions as well as by substantial heterogeneity in protocols and outcome measures. Among prior digital cognitive rehabilitation trials in MCI, Graessel and colleagues [76] delivered a 12-month, individualized, home-based, self-administered multidomain computerized training, reporting larger gains in global cognitive functioning in the individualized arm than in a basic, non-individualized active comparator. Park et al. [77], adopting a home-based multidomain intervention, reported improvements in cognition and depressive symptoms in amnestic MCI that were maintained at three-month follow-up. Conversely, the home-based multidomain program by Duff et al. [78], did not find superiority over a non-progressive active comparator on standardized neuropsychological measures. Overall, these and related studies [59,79,80,81] enrolled larger samples, relied on clinical-setting delivery, and employed usual-care or passive comparators. Only a minority adopted active control conditions or medium- to long-term follow-ups. In this regard, this study presents at least two distinctive features, even though it is important to consider the limitations listed in the next section. First, the current intervention—like that of Graessel et al. [76]—was administered via a tablet-based, pre-configured device specifically intended to minimize technical demands, in contrast to previous home-based programs that were typically delivered through personal computers or general-purpose computerized platforms [78]. While this choice does not by itself address the digital-literacy barriers reported in older adults [59,64,65], it represents a step toward broader accessibility in home-based digital rehabilitation. Second, in line with recent demands to expand the outcome framework for cognitive rehabilitation in early decline [29,30,31], this pilot study combined standardized neuropsychological and psychological assessments with training-derived performance metrics extracted directly from the platform. This multidimensional approach, increasingly promoted in recent years [82], enabled the detection of session-related changes that would have remained invisible to standardized batteries alone.

### 4.2. Strengths, Limitations, and Future Directions

The present study has several strengths. First, it is embedded within the broader MASCoD clinical research program, which provides a theoretically grounded framework for the investigation [7,35]. Additionally, it benefits from a thorough multidimensional assessment in line with contemporary best practices [38]. Second, combining standardized measurements with training-derived performance metrics provides a complementary lens that can capture within-person changes that are frequently missed by traditional instruments [20,23,32]. Third, an individualized analytical approach based on within-participant regression models preserved person-specific trajectories, which are especially useful in a heterogeneous population since group-level studies may mask relevant effects [42,43]. Fourth, the home-based delivery format improves ecological validity by more precisely replicating the real-world situations under which people would participate in cognitive training.

Besides strengths, there are limits that need to be considered. First, despite the adoption of a randomization strategy, baseline features were not balanced between groups, and the limited sample size significantly reduced statistical power. Since these features are known to affect cognitive functioning and rehabilitative participation, they make it more difficult to interpret between-group comparisons [61]. In particular, the age difference between groups is clinically meaningful, and although raw cognitive scores were corrected for age according to Italian normative data, normative correction does not fully control for age-related differences in neuroplasticity and responsiveness to repeated practice. Relatedly, the present study did not include biological or mechanistic correlates of neuroplasticity, which would have allowed a more direct assessment of the neural processes potentially underlying the observed cognitive trajectories and strengthened the translational relevance of the findings. Second, the clinical heterogeneity of MCI is itself a limitation, because the sample size did not allow stratified analysis according to MCI subtype or impaired cognitive domain. Third, the absence of an active control condition limits the possibility to attribute observed changes specifically to the cognitive training content. Therefore, nonspecific factors such as repeated contact with researchers, expectations of benefit, familiarity with the testing procedure, and placebo-related mechanisms cannot be ruled out. Fourth, baseline digital literacy was not assessed. This is relevant because participants’ familiarity with digital devices, confidence in using technology, and perceived autonomy in managing the platform may have influenced adherence to the home-based component of the intervention and, more broadly, the rehabilitative outcomes. Fifth, the study did not include an assessment of the engagement with the digital rehabilitation program. Although adherence was quantified, this measure alone does not capture how participants experienced or interacted with the intervention. Sixth, while the home-based delivery format was specifically chosen to enhance the ecological validity of the intervention, the outcome framework itself remained limited in this respect: both the standardized neuropsychological battery and the Neurotablet^®^ tasks assess cognition under structured conditions that do not directly capture the demands of everyday functioning. Finally, the lack of intermediate assessment time points may have reduced the ability to detect the temporal dynamics of intervention effects [24].

Future studies should address the limitations through several complementary strategies. First, larger randomized controlled trials with an active control condition are required to distinguish specific training effects from non-specific or placebo-related contributions and provide more convincing proof of efficacy [21,24]. Second, even in small-sample designs, stratified randomization on key prognostic variables—particularly age, given its influence on cognitive performance and on neuroplasticity—would ensure baseline comparability between groups. Third, the integration of neuroplasticity correlates in future trials would help clarify the neural mechanisms potentially engaged by cognitive training and support the translational relevance of the findings. Fourth, future studies should include a baseline assessment of digital literacy and a more systematic monitoring of training engagement. Combining objective usage metrics with structured self-report and qualitative engagement measures would allow a more accurate examination of dose–response relationships and a better understanding of the factors that may promote engagement, adherence, and sustained use of the platform. Similarly, the inclusion of intermediate assessment time points in future studies would shed light on the temporal dynamics of training effects, helping enabling researchers to monitor how cognitive performance evolves throughout the intervention and to identify when potential benefits emerge, peak, or fade over time [10,19]. Lastly, the field continues to prioritize the development of increasingly sensitive outcome measures, such as training-derived composite indices and ecologically valid digital evaluations [83,84]. While comprehensive neuropsychological batteries are essential for diagnostic purposes and for assessing broader cognitive change, they may be insufficiently sensitive to detect subtle improvements in the specific processes targeted by the intervention. They should be complemented by more proximal outcome measures directly aligned with the cognitive processes and task demands targeted by the intervention. Future studies should therefore include training-specific measures selected according to the domains and exercises directly addressed by the platform. This approach would allow a clearer correspondence between the intervention content and the outcome measures, improving the ability to detect domain-specific treatment effects. Alongside this need for greater sensitivity, future research should also pursue greater ecological validity of the outcome measures, incorporating assessments capable of capturing cognitive functioning in conditions more representative of everyday life.

## 5. Conclusions

This pilot study investigated the effects of an individualized, home-based cognitive training program delivered via the Neurotablet^®^ platform in individuals with MCI. While standardized neuropsychological and psychological measures remained stable over time with no significant within- and between-group differences, training-derived performance metrics revealed heterogeneous individual trajectories, with session-related reductions in reaction times emerging in some participants—particularly in tasks involving attention and visuospatial planning. These findings suggest that repeated engagement with training tasks may be associated with measurable changes in task efficiency, even in the absence of detectable changes in standardized neuropsychological measures. Moreover, they highlight how digital training platforms can offer complementary, task-proximal indices of individual cognitive functioning. Despite important limitations—e.g., small sample size, baseline group imbalances, and the absence of direct adherence monitoring—these findings support the feasibility of individualized digital cognitive training for individuals with MCI and suggest that training-derived metrics deserve greater consideration as outcome measures in early cognitive rehabilitation. Larger, adequately powered trials with stratified randomization, active control conditions, extended follow-up, and comprehensive monitoring of training variables are needed to establish the clinical efficacy and specificity of such interventions.

## Figures and Tables

**Table 1:** Sociodemographic, clinical and lifestyle characteristics of the study sample.

Variables	Levels of the Variables	Total (n = 14)	EG (n = 7)	CG (n = 7)	*p*
Age *		69.5 (62.0, 78.0)	77.0 (72.5, 78.8)	62.0 (58.3, 66.8)	0.011
Years of schooling *		9.5 (8.0, 13.0)	10.0 (5.8, 13.0)	9.0 (8.0, 13.0)	0.74
Weight (kg) *		78.0 (63.8, 84.5)	72.0 (63.0, 89.0)	78.0 (67.5, 82.3)	0.60
Height (m) *		1.60 (1.57, 1.72)	1.66 (1.60, 1.73)	1.60 (1.51, 1.69)	0.59
BMI *		29.3 (23.4, 30.8)	28.1 (23.1, 29.4)	30.5 (24.2, 34.6)	0.29
Gender °	Male	10 (71.4%)	5 (71.4%)	5 (71.4%)	1
	Female	4 (28.6%)	2 (28.6%)	2 (28.6%)	
Marital status °	Married/Cohabiting	10 (71.4%)	3 (42.9%)	7 (100%)	0.07
	Widowed	4 (28.6%)	4 (57.1%)	0 (0%)	
Education °	Elementary school	2 (14.3%)	2 (28.6%)	0 (0%)	0.37
	Junior high school	4 (28.6%)	1 (14.3%)	3 (42.9%)	
	High school	7 (50.0%)	4 (57.1%)	3 (42.9%)	
	Bachelor’s degree	0 (0%)	0 (0%)	0 (0%)	
	Master’s degree	0 (0%)	0 (0%)	0 (0%)	
	Postgraduate	1 (7.1%)	0 (0%)	1 (14.3%)	
Employment Status °	Full-time employee	3 (21.4%)	0 (0%)	3 (42.9%)	0.19
	Retired	11 (78.6%)	7 (100%)	4 (57.1%)	
Social and Family Support °	Spouse/Partner	8 (57.1%)	2 (28.6%)	6 (85.7%)	0.10
	Son/Daughter	6 (42.9%)	5 (71.4%)	1 (14.3%)	
Smoking °	Yes	3 (21.4%)	2 (28.6%)	1 (14.3%)	0.63
	No	8 (57.1%)	3 (42.9%)	5 (71.4%)	
	Former smoker	3 (21.4%)	2 (28.6%)	1 (14.3%)	
Physical Activity °	Yes	7 (53.8%)	4 (66.7%)	3 (42.9%)	0.59
	No	6 (46.2%)	2 (33.3%)	4 (57.1%)	
Overweight °	Yes	6 (42.9%)	2 (28.6%)	4 (57.1%)	0.59
	No	8 (57.1%)	5 (71.4%)	3 (42.9%)	
Alcohol °	Yes	1 (7.1%)	1 (14.3%)	0 (0%)	1
	No	13 (92.9%)	6 (85.7%)	7 (100%)	
Drug Use History °	Yes	0 (0%)	0 (0%)	0 (0%)	-
	No	14 (100%)	7 (100%)	7 (100%)	
Family History of NCDs °	Yes	7 (53.8%)	3 (50.0%)	4 (57.1%)	1
	No	6 (46.2%)	3 (50.0%)	3 (42.9%)	
Diabetes °	Yes	2 (14.3%)	0 (0%)	2 (28.6%)	0.46
	No	12 (85.7%)	7 (100%)	5 (71.4%)	
Hyperuricemia °	Yes	0 (0%)	0 (0%)	0 (0%)	-
	No	14 (100%)	7 (100%)	7 (100%)	
Hypertension °	Yes	7 (50.0%)	3 (42.9%)	4 (57.1%)	1
	No	7 (50.0%)	4 (57.1%)	3 (42.9%)	
Dyslipidemia °	Yes	1 (7.1%)	0 (0%)	1 (14.3%)	1
	No	13 (92.9%)	7 (100%)	6 (85.7%)	

Notes. * For continuous variables data are reported as Median (Q_1_–Q_3_); ° For categorical variables data are reported as n (%). *p*-values are from chi-squared test or Fisher’s exact test and Mann–Whitney U test for categorical and continuous variables, respectively.

**Table 2:** Individual adherence to the home-based digital cognitive rehabilitation protocol.

Participant	Training Time (Minutes)	Adherence (%) *
EG 1	1589.4	132.4
EG 2	842.9	70.2
EG 3	2569.0	214.1
EG 4	825.8	68.8
EG 5	1344.1	112.0
EG 6	429.3	35.8
EG 7	856.5	71.4

Notes. * Adherence was calculated as the ratio between the actual completed training time and the expected training time, expressed as a percentage. Values above 100% indicate completion of training time exceeding the expected dose.

**Table 3:** (**a**) Descriptive statistics of within-group changes. (**b**) Effect of the intervention (EG) over time versus waiting-list (CG).

(**a**)								
**Variables**	**EG (n = 7)**				**CG (n = 7)**			
	**T_0_**	**T_1_**	**Delta EG**	**EG RBC**	**T_0_**	**T_1_**	**Delta CG**	**CG RBC**
MMSE	27.72 (26.73, 29.36)	27.47 (26.86, 28.06)	0.00 (−1.71, 0.65)	0.3	29.08 (27.79, 29.51)	28.61 (28.39, 29.25)	−0.94 (−0.95, 1.05)	0.1
ACE-III	85.18 (79.52, 86.80)	85.03 (81.57, 85.73)	2.50 (−4.75, 5.04)	−0.2	75.75 (74.78, 81.78)	77.63 (71.13, 85.78)	−0.25 (−2.50, 4.00)	−0.1
FAB	12.44 (11.77, 14.91)	14.60 (14.23, 16.28)	0.08 (−0.72, 3.75)	−0.3	13.15 (11.33, 13.33)	14.24 (11.24, 15.93)	1.05 (0.02, 2.61)	0.4
TMT-A	33.82 (30.31, 49.08)	25.16 (23.69, 34.78)	−9.80 (−14.54, −7.33)	0.9	38.30 (30.14, 41.97)	42.76 (32.46, 57.81)	4.95 (−3.56, 16.24)	0.4
TMT-B	110.63 (66.27, 195.15)	79.97 (73.96, 106.54)	−30.76 (−113.00, 22.99)	0.5	100.75 (79.91, 128.52)	133.66 (112.88, 167.54)	20.28 (−21.32, 60.13)	0.4
Stroop time	15.88 (13.81, 17.32)	18.81 (11.93, 25.65)	0.47 (−3.09, 13.17)	−0.3	21.94 (18.30, 25.68)	23.89 (13.19, 24.91)	0.79 (−1.79, 5.59)	0.1
Stroop errors	0.00 (0.00, 0.00)	0.02 (0.00, 3.76)	0.02 (0.00, 3.76)	−1.0	0.00 (0.00, 1.03)	0.00 (0.00, 0.97)	0.00 (0.00, 0.00)	0.3
DSF	5.19 (4.58, 5.36)	5.58 (4.71, 6.32)	0.98 (−0.74, 1.77)	−0.4	4.69 (4.31, 5.59)	4.78 (4.15, 5.80)	0.02 (−0.96, 0.79)	0.1
DSB	4.20 (3.65, 5.10)	4.16 (3.79, 4.33)	0.00 (−0.73, 0.76)	−0.1	3.63 (2.94, 4.12)	4.06 (2.34, 4.28)	0.02 (−1.47, 0.91)	0.1
CSF	4.62 (4.30, 5.22)	5.62 (4.58, 6.90)	1.00 (−0.48, 1.78)	−0.7	4.76 (4.25, 5.22)	5.27 (4.18, 5.74)	0.17 (0.04, 1.03)	0.7
CSB	4.22 (3.45, 4.79)	4.69 (3.87, 5.36)	0.06 (−0.71, 2.53)	−0.4	3.64 (2.46, 4.80)	3.83 (3.09, 4.96)	1.07 (−1.73, 2.08)	0.2
RAVLT immediate recall	38.64 (35.12, 40.74)	34.50 (32.46, 54.74)	1.66 (−6.56, 14.00)	−0.3	38.98 (35.98, 39.66)	39.97 (37.33, 46.52)	1.00 (0.44, 7.73)	0.6
RAVLT delayed recall	6.19 (4.62, 10.66)	6.76 (0.00, 12.47)	0.50 (−0.85, 1.99)	−0.2	7.86 (5.08, 9.23)	4.93 (4.03, 14.48)	−0.89 (−1.78, 5.25)	−0.1
ROCF recall	10.62 (10.09, 15.77)	11.86 (9.15, 22.25)	0.00 (−1.91, 8.05)	−0.2	13.84 (6.76, 17.34)	10.43 (9.22, 14.26)	−1.28 (−4.50, 4.34)	−0.1
ROCF copy	30.68 (26.04, 32.94)	32.33 (31.78, 35.31)	1.92 (−1.16, 5.13)	−0.4	28.31 (25.29, 32.47)	29.81 (23.49, 32.59)	0.09 (−4.39, 6.20)	0.1
CDT	57.55 (53.22, 60.33)	57.26 (54.05, 57.98)	−2.96 (−3.88, 5.02)	0.1	56.35 (52.00, 59.86)	55.50 (52.67, 56.91)	−0.83 (−2.34, 3.98)	−0.1
Phonemic Fluency	40.96 (29.43, 47.33)	38.97 (26.27, 45.46)	3.00 (−8.25, 5.75)	−0.1	37.75 (28.60, 41.21)	36.08 (22.60, 45.26)	−3.00 (−10.75, 10.00)	−0.2
Semantic Fluency	44.15 (38.09, 48.05)	40.35 (36.13, 46.05)	0.20 (−6.30, 0.90)	0.2	38.74 (36.30, 53.99)	40.75 (36.20, 52.53)	0.40 (−11.15, 4.70)	−0.1
PHQ-9	5.00 (3.50, 11.00)	8.00 (3.75, 15.00)	0.00 (−4.00, 2.50)	0.1	11.00 (2.50, 12.00)	7.00 (1.50, 9.50)	−3.00 (−5.00, 2.50)	−0.3
GAD-7	5.00 (2.50, 12.00)	13.00 (4.75, 16.00)	2.00 (1.00, 7.25)	−0.7	6.00 (4.00, 9.00)	5.00 (0.25, 12.75)	−2.00 (−7.50, 1.50)	−0.3
CFI	5.50 (3.00, 9.00)	4.00 (2.00, 6.00)	−1.25 (−5.50, 0.00)	0.4	6.00 (4.25, 6.75)	6.00 (2.00, 6.00)	0.00 (−0.75, 0.00)	−1.0
MASCoD	11.00 (10.00, 13.75)	12.00 (8.50, 15.00)	1.00 (−2.75, 1.75)	0.1	14.00 (8.75, 14.75)	12.00 (6.25, 15.50)	−1.00 (−1.75, 0.00)	−0.5
(**b**)								
**Variables**	**EG (n = 7)**		**CG (n = 7)**		***p*** **Group**	***p*** **Time**	***p*** **Group × Time**	
	**T_0_ EMM** **(95% CI)**	**T_1_ EMM** **(95% CI)**	**T_0_ EMM** **(95% CI)**	**T_1_ EMM** **(95% CI)**				
MMSE	28.1 (27.0, 29.2)	27.6 (26.5, 28.7)	28.8 (27.6, 29.9)	28.4 (27.3, 29.6)	0.1965 (1.87, 0.13)	0.3838 (0.82, 0.06)	0.8566 (0.03, 0.002)	
ACE-III	83.9 (76.7, 91.8)	85.9 (79.6, 92.7)	77.2 (71.1, 83.8)	78.4 (72.6, 84.6)	0.0474 (5.26, 0.30)	0.5811 (0.33, 0.03)	0.9195 (0.01, <0.001)	
FAB	13.4 (11.4, 15.8)	14.2 (12.1, 16.8)	13.0 (11.0, 15.3)	13.6 (11.5, 16.0)	0.6612 (0.2, 0.02)	0.3743 (0.85, 0.07)	0.9236 (0.01, <0.001)	
TMT-A	42.9 (27.1, 68.1)	33.2 (21.0, 52.7)	36.8 (23.2, 58.3)	42.4 (26.7, 67.2)	0.8766 (0.03, 0.002)	0.5877 (0.31, 0.03)	0.0789 (3.69, 0.24)	
TMT-B	149.4 (99.2, 225.0)	91.1 (58.6, 141.5)	105.5 (67.8, 164.2)	130.7 (84.0, 203.4)	0.9763 (0, 0)	0.4326 (0.67, 0.05)	0.0656 (4.27, 0.26)	
Stroop time	15.6 (11.5, 21.2)	18.2 (13.3, 24.7)	22.2 (16.3, 30.2)	22.0 (15.8, 30.6)	0.1145 (2.94, 0.20)	0.5830 (0.32, 0.03)	0.5401 (0.4, 0.03)	
Stroop errors	0.0 (−18.4, 18.4)	0.3 (−17.3, 17.9)	0.9 (−24.0, 25.8)	1.2 (−23.7, 26.1)	0.7658 (0.15, 0.01)	0.5335 (0.81, 0.06)	0.9608 (0, 0)	
DSF	5.2 (4.4, 6.0)	5.6 (4.8, 6.5)	4.9 (4.2, 5.7)	4.8 (4.1, 5.6)	0.2154 (1.71, 0.13)	0.6486 (0.22, 0.02)	0.4263 (0.68, 0.05)	
DSB	4.3 (3.6, 5.2)	4.3 (3.6, 5.2)	3.5 (2.9, 4.2)	3.7 (3.0, 4.5)	0.0668 (4.14, 0.26)	0.7150 (0.14, 0.01)	0.7405 (0.12, 0.01)	
CSF	4.7 (4.1, 5.5)	5.6 (4.8, 6.5)	4.6 (4.0, 5.4)	5.0 (4.3, 5.8)	0.3857 (0.81, 0.04)	0.0754 (3.79, 0.24)	0.4763 (0.54, 0.04)	
CSB	4.5 (3.4, 5.9)	4.6 (3.6, 6.0)	3.9 (2.9, 5.2)	4.2 (3.2, 5.6)	0.3945 (0.80, 0.06)	0.6268 (0.25, 0.02)	0.8358 (0.05, 0.004)	
RAVLT immediate recall	38.1 (30.9, 46.9)	40.3 (32.7, 49.7)	36.0 (29.7, 43.7)	38.6 (31.5, 47.3)	0.6748 (0.19, 0.02)	0.2938 (1.23, 0.09)	0.9194 (0.01, <0.001)	
RAVLT delayed recall	7.5 (4.4, 12.7)	9.1 (5.1, 16.2)	6.7 (4.3, 10.4)	6.6 (4.1, 10.6)	0.4338 (0.68, 0.05)	0.6053 (0.29, 0.02)	0.5700 (0.35, 0.03)	
ROCF recall	12.2 (8.5, 17.4)	14.1 (9.8, 20.1)	11.5 (8.1, 16.5)	11.5 (8.1, 16.5)	0.5466 (0.38, 0.03)	0.5357 (0.41, 0.03)	0.5342 (0.41, 0.03)	
ROCF copy	28.5 (23.4, 34.6)	29.7 (24.4, 36.1)	28.2 (23.2, 34.3)	28.0 (23.0, 34.0)	0.7571 (0.10, 0.008)	0.8053 (0.06, 0.005)	0.7221 (0.13, 0.01)	
CDT	54.2 (47.3, 62.0)	54.8 (47.9, 62.7)	52.1 (45.6, 59.7)	53.7 (47.0, 61.5)	0.6969 (0.16, 0.013)	0.6826 (0.18, 0.015)	0.8537 (0.04, 0.003)	
Phonemic Fluency	36.7 (26.8, 50.1)	36.7 (26.8, 50.1)	35.7 (26.2, 48.8)	32.0 (23.5, 43.8)	0.6472 (0.22, 0.018)	0.6280 (0.25, 0.02)	0.6250 (0.25, 0.02)	
Semantic Fluency	43.2 (36.4, 51.2)	41.2 (34.7, 48.9)	43.7 (36.9, 51.9)	41.6 (35.1, 49.3)	0.9121 (0.01, <0.001)	0.3673 (0.88, 0.07)	0.9758 (0, 0)	
PHQ-9	7.8 (3.8, 16.0)	8.7 (4.2, 17.8)	7.0 (3.6, 13.8)	5.3 (2.7, 10.3)	0.4392 (0.65, 0.05)	0.7012 (0.16, 0.013)	0.4099 (0.74, 0.06)	
GAD-7	6.5 (3.4, 12.6)	10.4 (5.4, 20.0)	6.7 (3.6, 12.5)	7.7 (3.8, 15.5)	0.7111 (0.15, 0.01)	0.1654 (2.33, 0.16)	0.4383 (0.67, 0.05)	
CFI	5.6 (2.9, 10.9)	4.0 (2.1, 7.9)	4.9 (2.6, 9.0)	4.1 (2.2, 7.6)	0.8612 (0.03, 0.002)	0.2008 (1.85, 0.13)	0.6865 (0.17, 0.014)	
MASCoD	11.9 (8.8, 16.1)	11.3 (8.3, 15.3)	11.7 (8.7, 15.9)	10.3 (7.6, 14.0)	0.7805 (0.08, 0.007)	0.2215 (1.66, 0.12)	0.5922 (0.30, 0.02)	

Notes. (a). Within-group changes are expressed as median and interquartile range (IQR). Within-group changes (Delta) were computed as paired differences (T1–T0). Effect sizes for within-group changes were estimated using the median of paired differences and the rank-biserial correlation (RBC). (b). Estimated marginal means (EMM) with 95% confidence intervals were estimated from generalized linear mixed models (GLMM) and back-transformed to the original scale. For each outcome, Type III tests of fixed effects are reported with numerator and denominator degrees of freedom (df = 1, 12). *p*-values for main effects of group and time and group × time interaction are reported with (F statistics, partial eta squared). Partial eta squared (η^2^p) is reported as an effect size measure and was calculated from the F statistics.

**Table 4:** Individual MCI subtype profiles at T_0_ and T_1_ and descriptive longitudinal trajectories.

Participant	T_0_ MCI Subtype	T_1_ MCI Subtype	Trajectory
EG 1	na-MCI multidomain (visuospatial long-term memory; visuospatial working memory; frontal executive functions; visuo-constructive and visuospatial abilities)	na-MCI multidomain (verbal short-term memory; visual-spatial and planning skills)	Same MCI subtype with reduction in deficits
EG 2	na-MCI multidomain (verbal short-term memory; visuospatial working memory; frontal executive functions; alternating attention; visuo-constructive and visuospatial abilities)	No formal MCI	Reduction in deficits
EG 3	a-MCI multidomain (verbal learning abilities; verbal long-term memory; visuospatial long-term memory; verbal short-term memory; frontal executive functions; selective attention and visuospatial search; alternating attention; inhibition and sensitivity to interference; visuo-constructive and visuospatial abilities; visual-spatial and planning skills; phonemic fluency)	a-MCI multidomain (verbal learning abilities; verbal long-term memory; visuospatial long-term memory; visuospatial short-term memory; frontal executive functions; selective attention and visuospatial search; inhibition and sensitivity to interference; visuo-constructive and visuospatial abilities)	Same MCI subtype with progression in deficits
EG 4	a-MCI multidomain (verbal long-term memory; visuospatial long-term memory; visuospatial short-term memory)	a-MCI single-domain (verbal learning abilities; verbal long-term memory; visuospatial long-term memory)	Same MCI subtype with reduction in deficits
EG 5	na-MCI multidomain (visuospatial long-term memory; verbal short-term memory; frontal executive functions)	na-MCI single-domain (visuospatial long-term memory)	Same MCI subtype with reduction in deficits
EG 6	a-MCI multidomain (verbal learning abilities; verbal long-term memory; visuospatial short-term memory)	na-MCI single-domain (visuospatial working memory)	MCI shift with reduction in deficits
EG 7	a-MCI multidomain (verbal long-term memory; alternating attention)	a-MCI multidomain (verbal long-term memory; inhibition and sensitivity to interference)	Same MCI subtype with progression in deficits
CG 1	na-MCI multidomain (verbal short-term memory; visuospatial short-term memory; visuospatial working memory; frontal executive functions)	a-MCI multidomain (verbal long-term memory; verbal short-term memory; visuospatial short-term memory; verbal working memory; visuospatial working memory; frontal executive functions; visuo-constructive and visuospatial abilities; phonemic fluency; semantic fluency)	MCI shift with severe progression
CG 2	na-MCI multidomain (frontal executive functions; visuo-constructive and visuospatial abilities)	na-MCI multidomain (visuospatial long-term memory; visuo-constructive and visuospatial abilities)	Same MCI subtype with profile reorganization
CG 3	a-MCI multidomain (verbal learning abilities; verbal long-term memory; visuospatial long-term memory; visuospatial short-term memory; visuospatial working memory; frontal executive functions; visuo-constructive and visuospatial abilities)	a-MCI multidomain (verbal learning abilities; verbal long-term memory; visuospatial long-term memory; verbal short-term memory; visuospatial short-term memory; visuospatial working memory; alternating attention; inhibition and sensitivity to interference)	Same MCI subtype with reduction in deficits
CG 4	na-MCI multidomain (verbal short-term memory; verbal working memory; visuospatial working memory; frontal executive functions; visuo-constructive and visuospatial abilities)	na-MCI multidomain (verbal short-term memory; visuo-constructive and visuospatial abilities)	Same MCI subtype with reduction in deficits
CG 5	a-MCI multidomain (verbal long-term memory; verbal working memory)	a-MCI multidomain (verbal learning abilities; verbal long-term memory; verbal working memory)	Same MCI subtype with progression in deficits
CG 6	na-MCI multidomain (visuospatial long-term memory; verbal short-term memory; frontal executive functions; inhibition and sensitivity to interference; visuo-constructive and visuospatial abilities; visual-spatial and planning skills)	a-MCI multidomain (verbal long-term memory; visuospatial long-term memory; verbal working memory; visuospatial working memory; frontal executive functions; selective attention and visuospatial search; inhibition and sensitivity to interference; visuo-constructive and visuospatial abilities; visual-spatial and planning skills; phonemic fluency)	MCI shift with severe progression
CG 7	a-MCI multidomain (verbal long-term memory; visuospatial long-term memory; frontal executive functions; visuo-constructive and visuospatial abilities)	a-MCI multidomain (verbal long-term memory; visuospatial long-term memory; visuospatial short-term memory; visuo-constructive and visuospatial abilities)	Same MCI subtype with progression in deficits

## Data Availability

Data are available from the corresponding author upon request due to privacy and ethical restrictions.

## Supplementary Materials

The following supporting information can be downloaded at: https://www.mdpi.com/article/10.3390/brainsci16060582/s1, File S1: Detailed list of neuropsychological and psychological outcome measures; File S2: Neurotablet^®^ training outputs; File S3: Radar Plots.
