# Supplementary material for: More than Just a Game: A Longitudinal Pilot Study on the Outcome Effects of Home-Based Digital Cognitive Rehabilitation in Outpatients with Mild Cognitive Impairment
Source: Brain Sci. 2026 May 29;16(6):582. doi: 10.3390/brainsci16060582 (PMC13297541; doi:10.3390/brainsci16060582)
Supplement: Supplementary file 1 [file brainsci-16-00582-s001.zip › brainsci-4309575-supplementary/Supplementary Materials/S1 - Detailed list of neuropsychological and psychological outcome measures.pdf]

Domains	Instrument	Functions assessed	Test structure	Scores Range, Interpretation and Normative Values*
Global cognition	Mini Mental State Examination (MMSE; Foderaro et al., 2022)	Global cognitive functioning	Structured interview composed of 11 tasks assessing temporal and spatial orientation, immediate and delayed recall, attention/calculation, language comprehension and production, and visuo-constructive copying	Raw scores range from 0 to 30. Higher scores indicate better performance. Adjusted scores ≥ 26.02 fall within the normal range
	Addenbrooke's Cognitive Examination III (ACE-III; Pigliautile et al., 2018)	Global cognitive functioning	Multidomain battery including tasks of orientation, learning and recall, verbal fluency, naming, comprehension, repetition, reading, writing, and visuospatial construction	Raw scores range from 0 to 100. Higher scores indicate better performance. Adjusted scores ≥ 68.68 fall within the normal range
Attention and executive functions	Frontal Assessment Battery (FAB; Aiello et al., 2021)	Main frontal executive functions	Six tasks assessing similarities, phonemic fluency, motor sequencing, conflicting instructions, go/no-go inhibition, and environmental autonomy	Raw scores range from 0 to 18. Higher scores indicate better performance. Adjusted scores ≥ 12.02 fall within the normal range
	Trail Making Test A (TMT-A; Siciliano et al., 2019)	Selective attention and visuospatial search	Sequential numeric connection task performed as quickly and accurately as possible	Score corresponds to completion time, with faster completion time indicative of a better performance. Adjusted scores ≤ 127 seconds fall within the normal range
	Trail Making Test B (TMT-B; Siciliano et al., 2019)	Alternating attention	It requires the alternating connection of numbers and letters in ascending/alphabetical order. The task should be performed as quickly and accurately as possible	Score corresponds to completion time, with faster completion time indicative of a better performance. Adjusted scores ≤ 294 seconds fall within the normal

Continued

Continued

	Stroop Test Time Effect (Caffarra et al., 2002b)	Inhibition and sensitivity to interference	Three conditions: word reading, color naming, and interference condition requiring inhibition of automatic responses	Score corresponds to completion time, with faster completion time indicative of a better performance. Adjusted scores ≤ 36.91 seconds fall within the normal range
	Stroop Test Errors Effect (Caffarra et al., 2002b)	Inhibition and sensitivity to interference		Score corresponds to the number of errors committed, with less error indicative of a better performance. Adjusted scores ≤ 4.23 fall within the normal range
Short-term memory	Digit Span Forward (DSF; Monaco et al., 2013)	Verbal short-term memory	Examiner reads sequences of digits of increasing length. Patient repeats each sequence in the same order	Raw scores range from 0 to 9. Higher scores indicate better performance. Adjusted scores ≥ 4.26 fall within the normal range
	Cubes' Span Forward (CSF; Monaco et al., 2013)	Visuospatial short-term memory	Examiner taps sequences of cubes of increasing length. Patient reproduces each sequence in the same order	Raw scores range from 0 to 9. Higher scores indicate better performance. Adjusted scores ≥ 3.46 fall within the normal range
Working memory	Digit Span Backward (DSB; Monaco et al., 2013)	Verbal working memory	Examiner reads sequences of digits of increasing length. Patient repeats each sequence in reverse order	Raw scores range from 0 to 8. Higher scores indicate better performance. Adjusted scores ≥ 2.65 fall within the normal range
	Cubes' Span Backward (CSB; Monaco et al., 2013)	Visuo-spatial working memory	Examiner taps sequences of cubes of increasing length. Patient reproduces each sequence in reverse order	Raw scores range from 0 to 8. Higher scores indicate better performance. Adjusted scores ≥ 3.17 fall within the normal range

Long-term memory	Rey–Osterrieth Complex Figure - Recall (ROCF Recall; Caffara et al., 2002a)	Visuospatial long-term memory	Delayed free recall of the ROCF after a retention interval	Raw scores range from 0 to 36. Higher scores indicate better performance. Adjusted scores ≥ 9.47 fall within the normal range
	Rey's Auditory Verbal Learning Test – Immediate Recall (RAVLT – Immediate Recall; Carlesimo et al., 1996)	Verbal learning abilities	Five learning trials of a 15-word list, with free recall after each trial. Total score is the sum of words recalled across all trials	Raw scores range from 0 to 75. Higher scores indicate better performance. Adjusted scores ≥ 28.53 fall within the normal range
	Rey's Auditory Verbal Learning Test – Delayed Recall (RAVLT – Delayed Recall; Carlesimo et al., 1996)	Long-term memory	Free recall of the 15-word list after a delay interval following the learning phase	Raw scores range from 0 to 15. Higher scores indicate better performance. Adjusted scores ≥ 4.69 fall within the normal range
Constructive praxis and visuospatial abilities	Rey–Osterrieth Complex Figure - Copy (ROCF Copy; Caffara, 2002a)	Visuo-constructive and visuospatial abilities	Free-hand copy of the ROCF	Raw scores range from 0 to 36. Higher scores indicate better performance. Adjusted scores ≥ 28.88 fall within the normal range
	Clock Drawing Test (CDT; Caffarra et al., 2011)	Visual-spatial and planning skills	Free-hand drawing of clock faces with numbers and hands set to a specified time	Raw scores range from 0 to 61. Higher scores indicate better performance. Adjusted scores ≥ 42.17 fall within the normal range
Language	Phonemic fluency test (Costa et al., 2014),	Phonemic fluency	Oral production of as many words as possible beginning with given letters (F, A, S), one minute per letter	Raw score corresponds to the number of words produced. Higher scores are indicative of a better performance. Adjusted scores ≥ 17.77 fall within the normal range

	Semantic fluency test (Costa et al., 2014)	Semantic fluency	Oral production of as many words as possible belonging to given semantic categories (colors, animals, fruits), one minute per category	Raw score corresponds to the number of words produced. Higher scores are indicative of a better performance. Adjusted scores ≥ 28.34 fall within the normal range
Psychological well-being	Patient Health Questionnaire-9 (PHQ-9) (Kroenke et al., 2001; Spitzer et al., 1999)	Depressive symptom severity	Self-report questionnaire with 9 items assessing the frequency of depressive symptoms over the past two weeks	Raw scores range from 0 to 27. Higher scores indicate more severe depressive symptoms. Scores of 5, 10, 15, and 20 represent cut points for mild, moderate, moderately severe, and severe depression, respectively
	Generalized Anxiety Disorder-7 (GAD-7) (Kroenke et al., 2007; Spitzer et al., 2006)	Anxiety symptom severity	Self-report questionnaire with 7 items assessing the frequency of anxiety symptoms over the past two weeks	Raw scores range from 0 to 21. Higher scores indicate more severe anxiety symptoms. Scores of 5, 10, and 15 represent cut points for mild, moderate, and severe anxiety, respectively
Subjective cognitive functioning	Cognitive Function Instrument (CFI, self-report) (Chipi et al., 2018)	Perceived cognitive change in daily activities	Self-report questionnaire with 14 items assessing perceived changes in cognitive and functional abilities in daily life	Higher scores indicate greater perceived cognitive decline
	Multidimensional Assessment of Subjective Cognitive Decline (MASCoD) (Maffoni et al., 2025; Maffoni et al., 2022)	Subjective cognition; risk factors; affective correlates	Brief multidimensional screening battery assessing subjective cognitive decline across three sections: comorbidities and risk factors, subjective cognition across multiple domains, and affective correlates	Higher scores indicate greater subjective cognitive complaints and associated risk

* Normative values are referred to Italian normative population.
