# Supplementary material for: More than Just a Game: A Longitudinal Pilot Study on the Outcome Effects of Home-Based Digital Cognitive Rehabilitation in Outpatients with Mild Cognitive Impairment
Source: Brain Sci. 2026 May 29;16(6):582. doi: 10.3390/brainsci16060582 (PMC13297541; doi:10.3390/brainsci16060582)
Supplement: Supplementary file 1 [file brainsci-16-00582-s001.zip › brainsci-4309575-supplementary/Supplementary Materials/S2 - Neurotablet® training outputs.pdf]

<table>
<tr><th>Patient</th><th>Multiple Alert</th><th>Regression results</th></tr>
<tr><td>1</td><td>

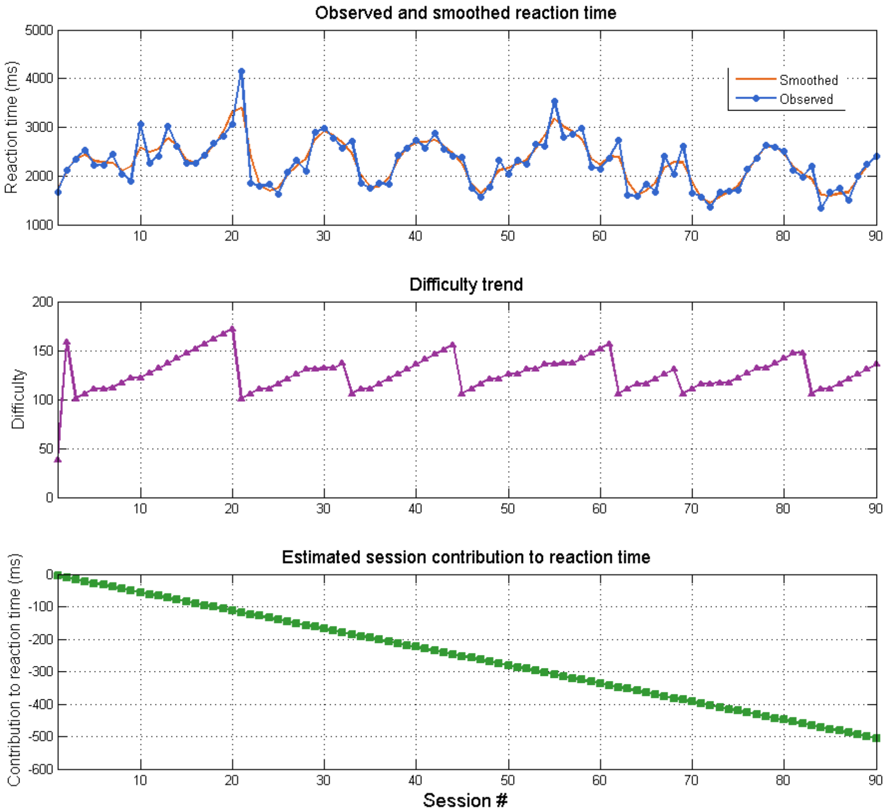

</td><td>

Estimated Coefficients:

	Estimate	SE	tStat	pValue
(Intercept)	1038	259.59	3.9985	0.00013332
Difficulty	11.676	1.987	5.876	7.5567e-08
Session (β_2)	-5.6147	1.4236	-3.9439	0.00016182

Number of observations: 90, Error degrees of freedom: 87
Root Mean Squared Error: 351
R-squared: 0.356, Adjusted R-Squared 0.342
F-statistic vs. constant model: 24.1, p-value = 4.71e-09

</td></tr>
</table>

<table>
<tr><th>Patient</th><th>Multiple Alert</th><th>Regression results</th></tr>
<tr>
<td>2</td>
<td>
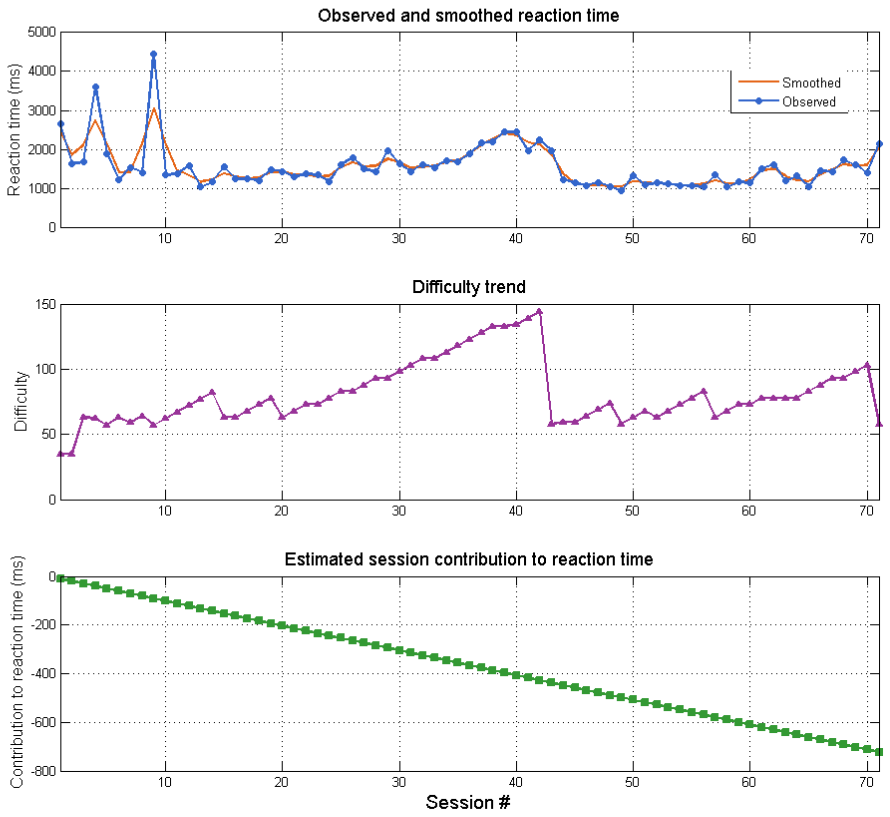

</td>
<td>
Estimated Coefficients:
<table>
<tr><th></th><th>Estimate</th><th>SE</th><th>tStat</th><th>pValue</th></tr>
<tr><td>(Intercept)</td><td>1340.2</td><td>166.54</td><td>8.0472</td><td>1.7986e-11</td></tr>
<tr><td>Difficulty</td><td>7.2094</td><td>1.9842</td><td>3.6334</td><td>0.00053835</td></tr>
<tr><td>Session (β_2)</td><td>-10.173</td><td>2.2891</td><td>-4.444</td><td>3.3403e-05</td></tr>
</table>
Number of observations: 71, Error degrees of freedom: 68
Root Mean Squared Error: 382
R-squared: 0.28, Adjusted R-Squared 0.259
F-statistic vs. constant model: 13.2, p-value = 1.39e-05
</td>
</tr>
</table>

<table>
<tr><th>Patient</th><th>Multiple Alert</th><th>Regression results</th></tr>
<tr><td>3</td><td>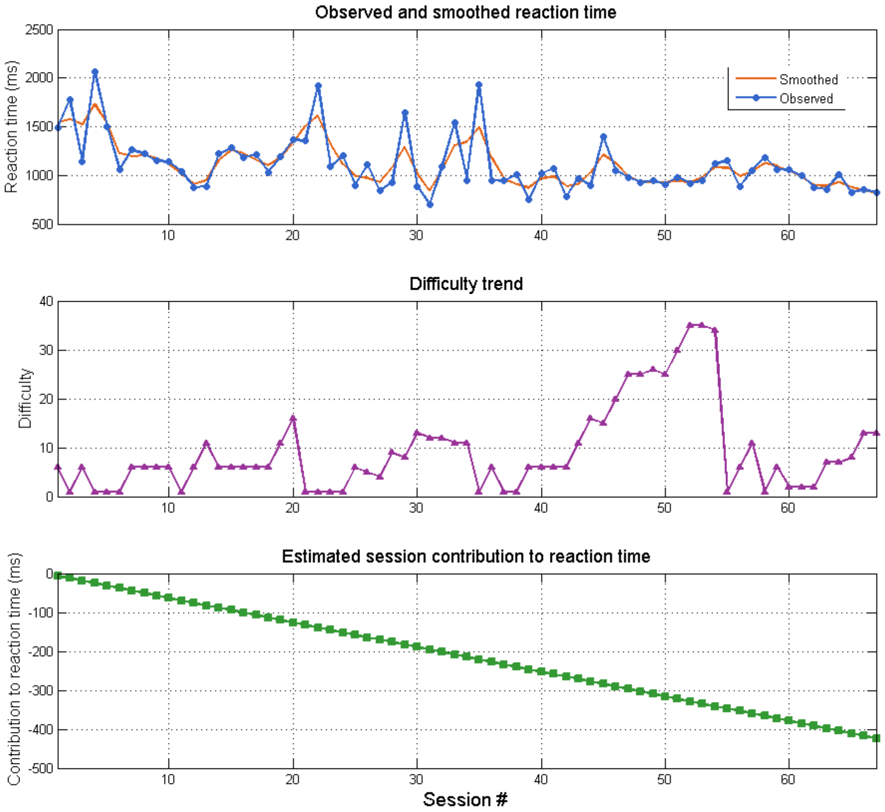
</td><td>Estimated Coefficients:

<table>
<tr><th></th><th>Estimate</th><th>SE</th><th>tStat</th><th>pValue</th></tr>
<tr><td>(Intercept)</td><td>1351.3</td><td>42.736</td><td>31.62</td><td>8.8694e-41</td></tr>
<tr><td>Difficulty</td><td>-2.8737</td><td>2.5555</td><td>-1.1245</td><td>0.265</td></tr>
<tr><td>Session (β_2)</td><td>-6.3162</td><td>1.1518</td><td>-5.4837</td><td>7.5665e-07</td></tr>
</table>
Number of observations: 67, Error degrees of freedom: 64
Root Mean Squared Error: 169
R-squared: 0.395, Adjusted R-Squared 0.376
F-statistic vs. constant model: 20.9, p-value = 1.04e-07</td></tr>
</table>

Patient	Multiple Alert	Regression results
4	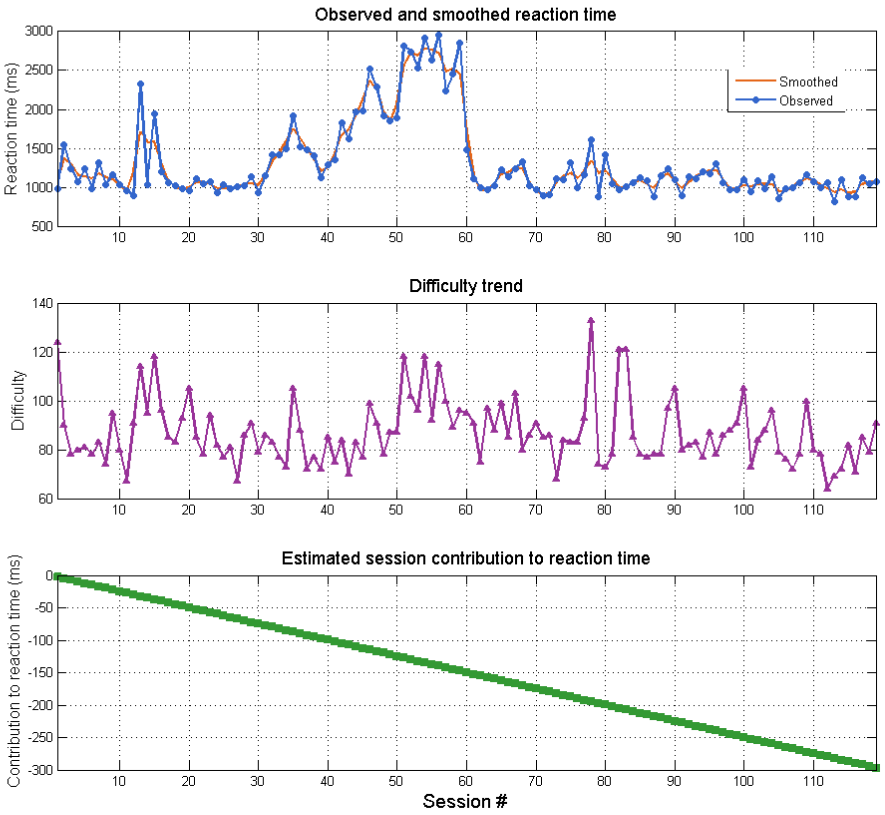	Estimated Coefficients:<br>Estimate SE tStat pValue<br>_________ _______ ________ ___________<br><br>(Intercept) 423.79 291.8 1.4523 0.14911<br>Difficulty 11.96 3.1221 3.8308 0.00020778<br>Session (β_2) -2.4982 1.1966 -2.0877 0.039013<br><br>Number of observations: 119, Error degrees of freedom: 116<br>Root Mean Squared Error: 445<br>R-squared: 0.155, Adjusted R-Squared 0.14<br>F-statistic vs. constant model: 10.6, p-value = 5.87e-05

Patient	Color Stroop	Regression results
5	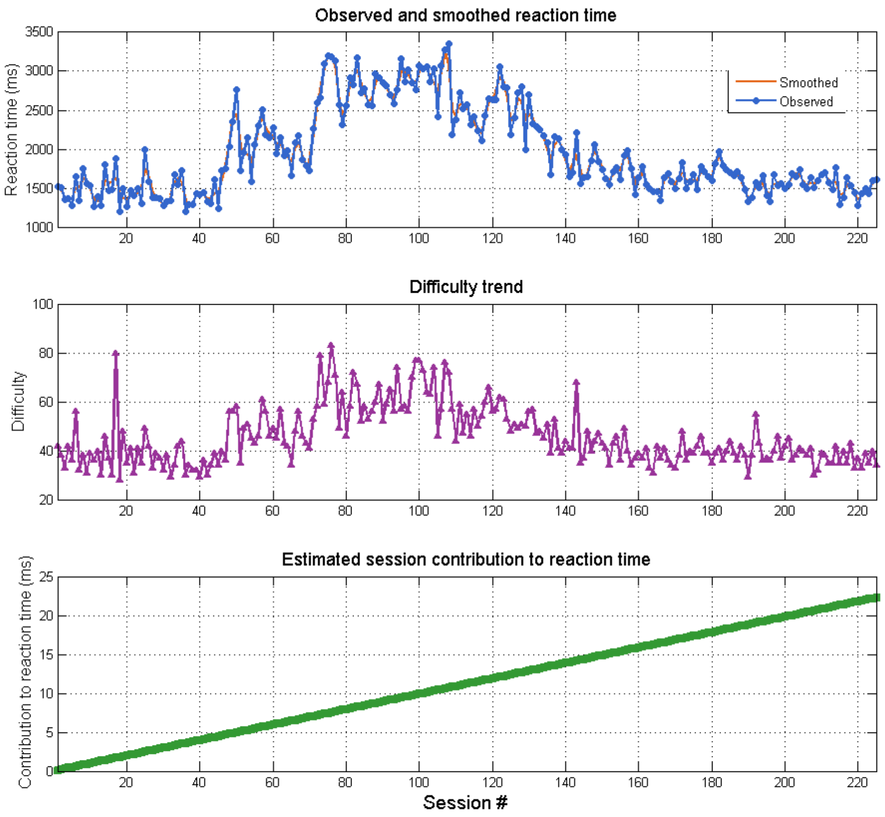	Estimated Coefficients:<br>Estimate SE tStat pValue<br>________ ________ _______ __________<br>(Intercept) 192.54 91.121 2.1131 0.035712<br>Difficulty 38.211 1.6852 22.674 1.0467e-59<br>Session (β_2) 0.098997 0.30639 0.3231 0.74692<br><br>Number of observations: 225, Error degrees of freedom: 222<br>Root Mean Squared Error: 295<br>R-squared: 0.702, Adjusted R-Squared 0.7<br>F-statistic vs. constant model: 262, p-value = 3.74e-59

<table>
<tr><th>Patient</th><th>Color Stroop</th><th>Regression results</th></tr>
<tr><td>1</td><td>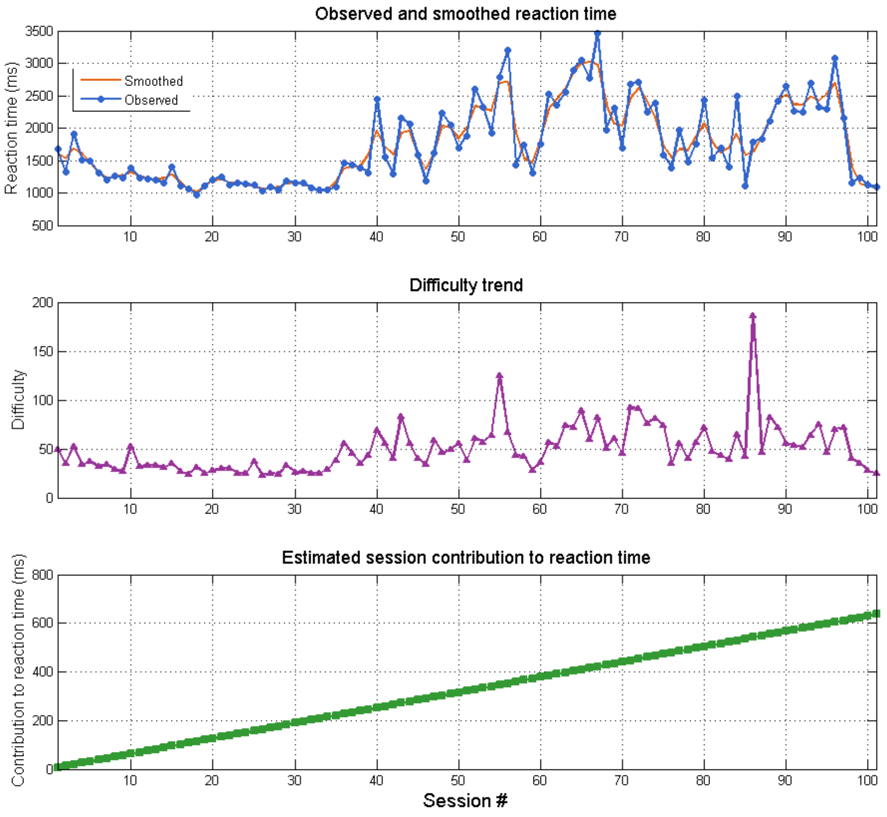
</td><td>Estimated Coefficients:
Estimate SE tStat pValue
_________ _______ _______ ___________
(Intercept) 836.83 94.127 8.8905 3.0877e-14
Difficulty 11.82 1.7966 6.5792 2.3332e-09
Session (β_2) 6.2971 1.4722 4.2772 4.4021e-05

Number of observations: 101, Error degrees of freedom: 98
Root Mean Squared Error: 383
R-squared: 0.53, Adjusted R-Squared 0.521
F-statistic vs. constant model: 55.3, p-value = 8.29e-17</td></tr>
</table>

Patient	Color Stroop	Regression results
6	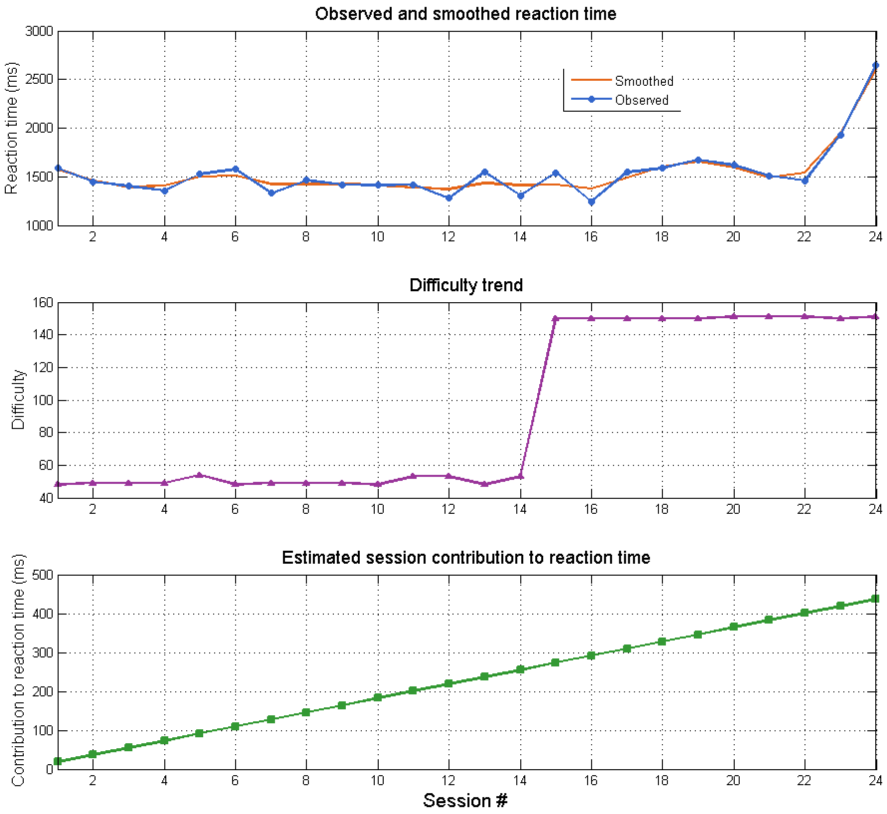	Estimated Coefficients:<br>Estimate SE tStat pValue<br>(Intercept) 1295.1 101.91 12.708 2.5115e-11<br>Difficulty 0.13155 1.8831 0.069858 0.94497<br>Session (β_2) 18.215 13.483 1.351 0.19108<br><br>Number of observations: 24, Error degrees of freedom: 21<br>Root Mean Squared Error: 233<br>R-squared: 0.268, Adjusted R-Squared 0.198<br>F-statistic vs. constant model: 3.85, p-value = 0.0377

<table>
<tr><th>Patient</th><th>Flow Free</th><th>Regression results</th></tr>
<tr><td>5</td><td>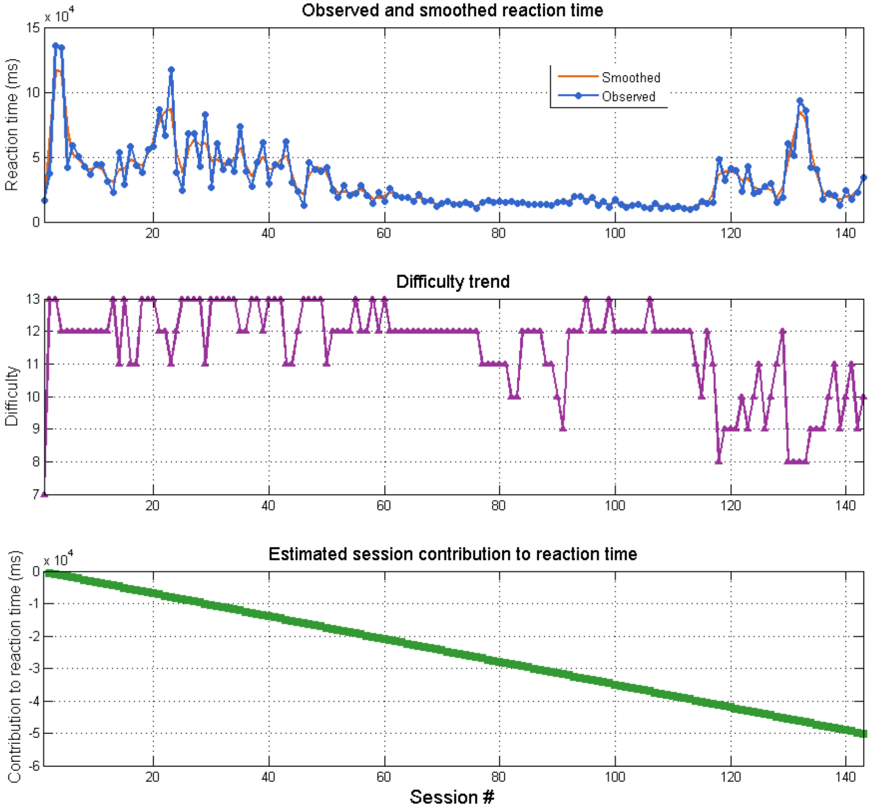
</td><td>Estimated Coefficients:
Estimate SE tStat pValue
(Intercept) 1.239e+05 16701 7.4183 1.0403e-11
Difficulty -5855.9 1279.1 -4.5781 1.0263e-05
Session (β_2) -351.12 42.076 -8.3449 6.1335e-14

Number of observations: 143, Error degrees of freedom: 140
Root Mean Squared Error: 1.71e+04
R-squared: 0.332, Adjusted R-Squared 0.323
F-statistic vs. constant model: 34.8, p-value = 5.28e-13</td></tr>
</table>

**Patient**	Flow Free	**Regression results**
1	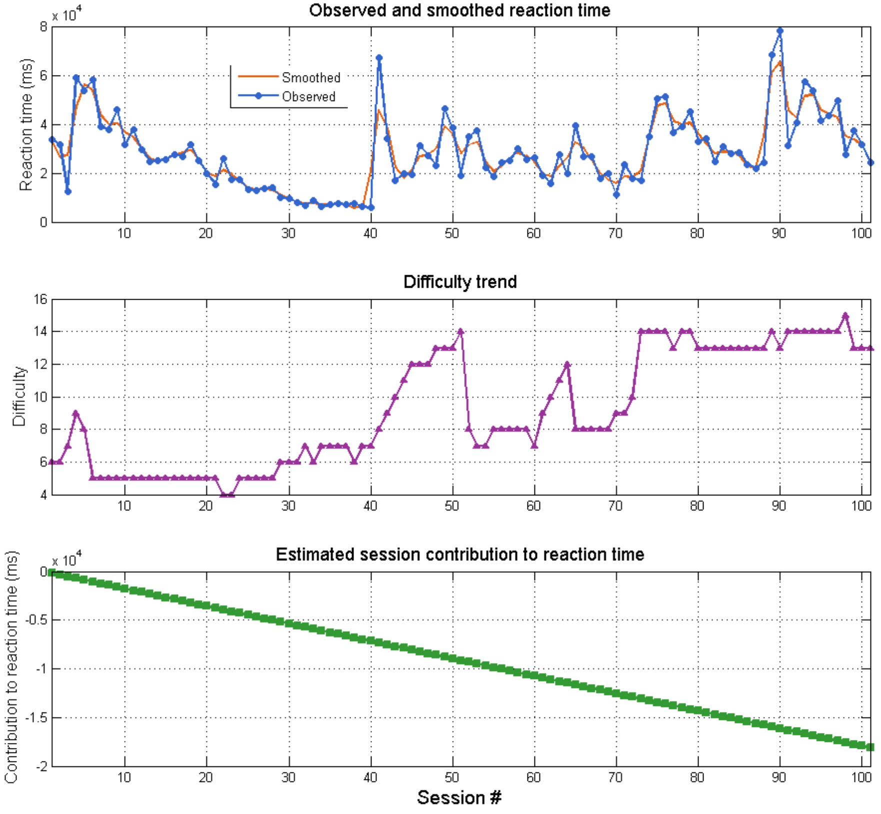	Estimated Coefficients:<br>Estimate SE tStat pValue<br>(Intercept) 10262 3326.2 3.0852 0.0026438<br>Difficulty 2974.5 611.51 4.8643 4.3849e-06<br>Session (β_2) -179.33 73.278 -2.4472 0.016177<br><br>Number of observations: 101, Error degrees of freedom: 98<br>Root Mean Squared Error: 1.13e+04<br>R-squared: 0.257, Adjusted R-Squared 0.242<br>F-statistic vs. constant model: 17, p-value = 4.67e-07

<table>
<tr><th>Patient</th><th>Flow Free</th><th>Regression results</th></tr>
<tr>
<td>3</td>
<td>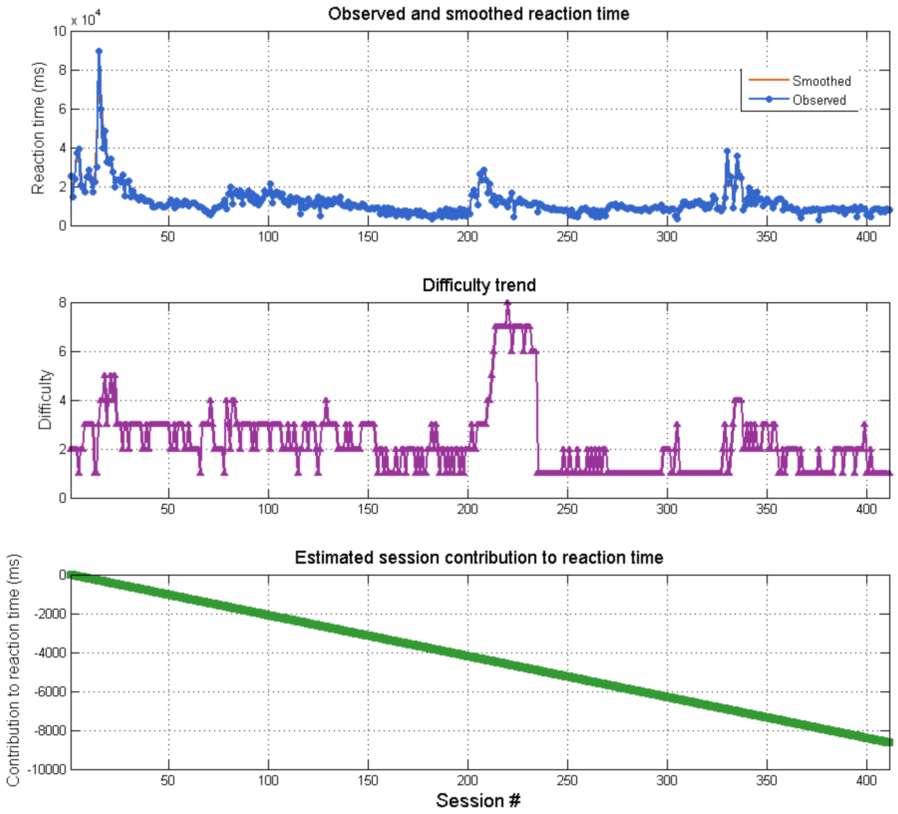
</td>
<td>Estimated Coefficients:
Estimate SE tStat pValue
________ ______ _______ __________

(Intercept) 14035 949.66 14.78 6.8437e-40
Difficulty 895.31 235.73 3.798 0.00016803
Session (β_2) -21.031 2.7944 -7.5259 3.3823e-13

Number of observations: 411, Error degrees of freedom: 408
Root Mean Squared Error: 6.41e+03
R-squared: 0.192, Adjusted R-Squared 0.188
F-statistic vs. constant model: 48.4, p-value = 1.39e-19</td>
</tr>
</table>

**Patient**	Flow Free	**Regression results**
6	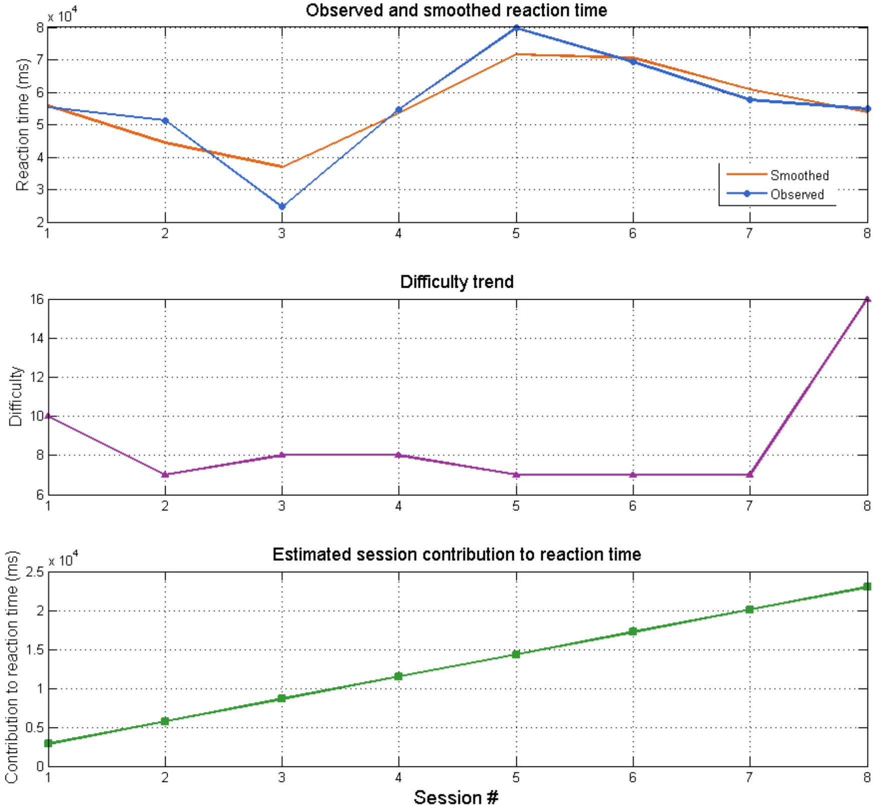	Estimated Coefficients:

	Estimate	SE	tStat	pValue
(Intercept)	55329	13466	4.1088	0.0092749
Difficulty	-1409.3	1497.7	-0.94102	0.3899
Session (β_2)	2870.9	1898.6	1.5121	0.19092

Number of observations: 8, Error degrees of freedom: 5
Root Mean Squared Error: 1.15e+04
R-squared: 0.331, Adjusted R-Squared 0.063
F-statistic vs. constant model: 1.24, p-value = 0.366

Patient	Flow Free	Regression results
4	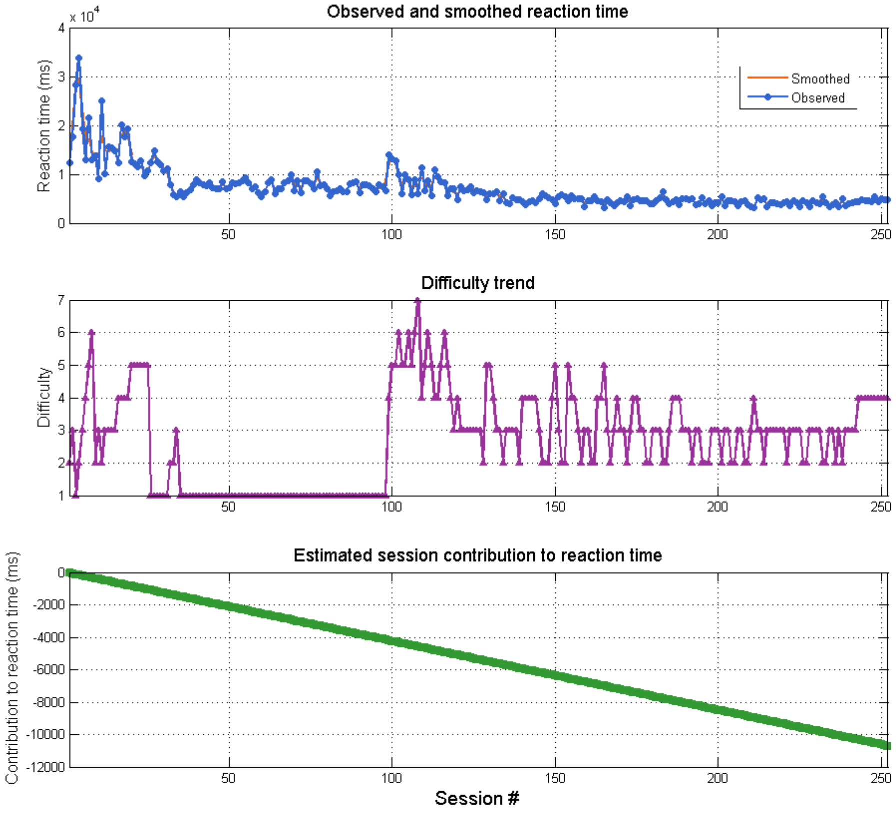	Estimated Coefficients:<br>Estimate SE tStat pValue<br>_________ _______ ________ ___________<br><br>(Intercept) 10939 395.82 27.636 8.0586e-78<br>Difficulty 570.43 116.34 4.9032 1.7024e-06<br>Session (β_2) -42.505 2.2351 -19.017 2.0571e-50<br><br>Number of observations: 252, Error degrees of freedom: 249<br>Root Mean Squared Error: 2.49e+03<br>R-squared: 0.592, Adjusted R-Squared 0.589<br>F-statistic vs. constant model: 181, p-value = 3.14e-49

<table>
<tr><th>Patient</th><th>GonoGo divided screen</th><th>Regression results</th></tr>
<tr><td>5</td><td>

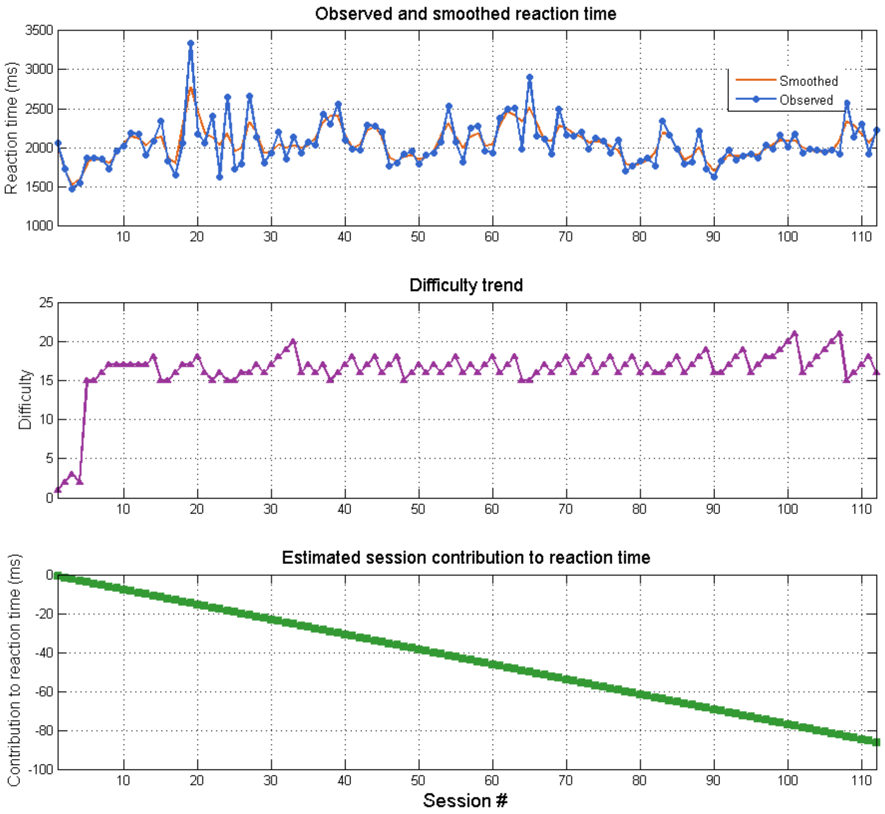

</td><td>

Estimated Coefficients:

	Estimate	SE	tStat	pValue
(Intercept)	1757.8	100.79	17.44	2.5655e-33
Difficulty	20.475	6.6524	3.0779	0.0026371
Session (β_2)	-0.77075	0.62648	-1.2303	0.22124

Number of observations: 112, Error degrees of freedom: 109
Root Mean Squared Error: 194
R-squared: 0.08, Adjusted R-Squared 0.0632
F-statistic vs. constant model: 4.74, p-value = 0.0106

</td></tr>
</table>

**Patient**	GonoGo divided screen	**Regression results**
1	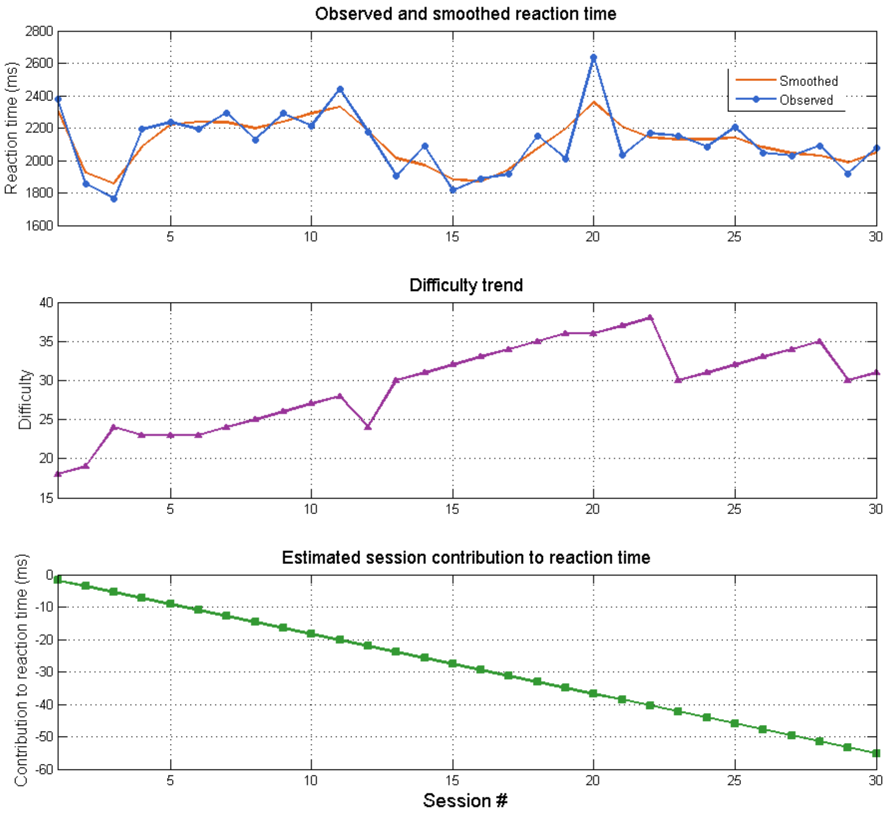	Estimated Coefficients:<br>Estimate SE tStat pValue<br>(Intercept) 2187.2 186.58 11.723 4.2286e-12<br>Difficulty -1.5668 8.2345 -0.19027 0.85052<br>Session (β_2) -1.8387 5.0913 -0.36113 0.72081<br><br>Number of observations: 30, Error degrees of freedom: 27<br>Root Mean Squared Error: 144<br>R-squared: 0.0282, Adjusted R-Squared -0.0438<br>F-statistic vs. constant model: 0.391, p-value = 0.68

**Patient**	GonoGo divided screen	**Regression results**
3	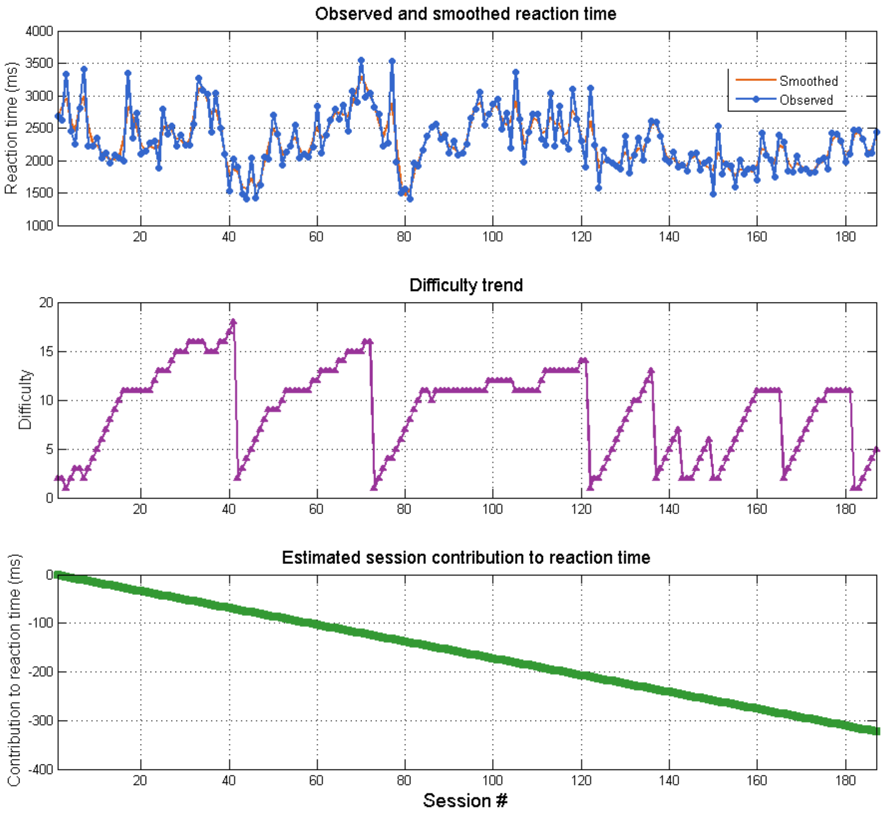 	Estimated Coefficients:<br>Estimate SE tStat pValue<br>(Intercept) 2242.5 79.8 28.101 1.7504e-68<br>Difficulty 23.361 5.7752 4.0451 7.6877e-05<br>Session (β_2) -1.7282 0.46743 -3.6972 0.0002875<br><br>Number of observations: 187, Error degrees of freedom: 184<br>Root Mean Squared Error: 335<br>R-squared: 0.177, Adjusted R-Squared 0.168<br>F-statistic vs. constant model: 19.8, p-value = 1.63e-08

<table>
<tr><th>Patient</th><th>GonoGo divided screen</th><th>Regression results</th></tr>
<tr><td>4</td><td>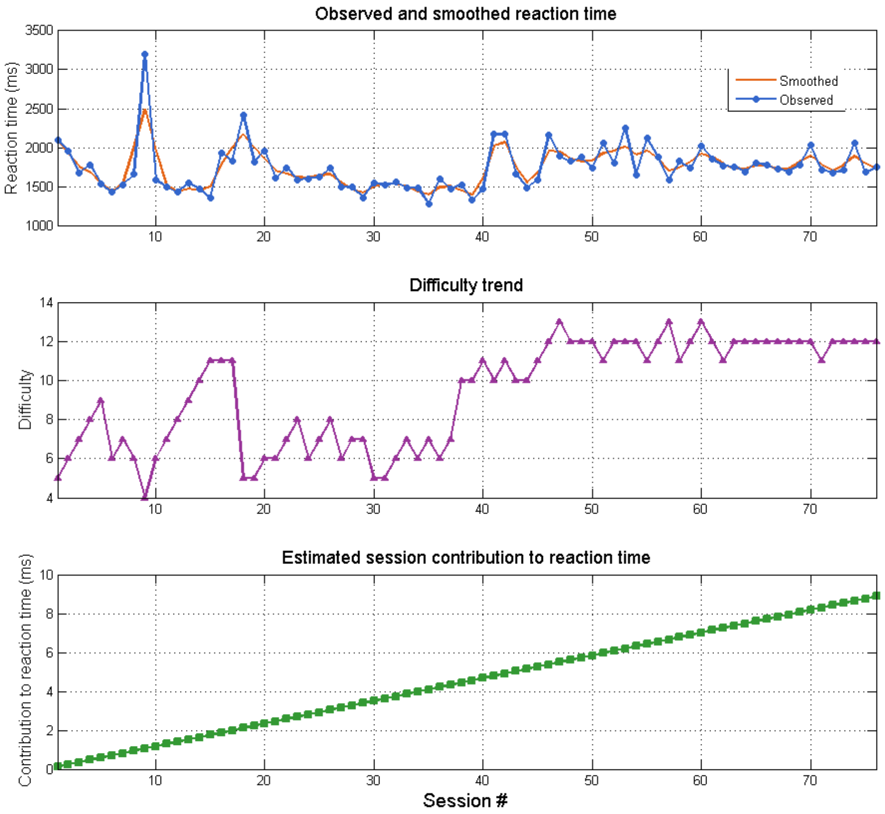
</td><td>Estimated coefficients:

<table>
<tr><th></th><th>Estimate</th><th>SE</th><th>tStat</th><th>pValue</th></tr>
<tr><td>(Intercept)</td><td>1599.1</td><td>96.54</td><td>16.564</td><td>1.9535e-26</td></tr>
<tr><td>Difficulty</td><td>14.259</td><td>14.455</td><td>0.98648</td><td>0.32715</td></tr>
<tr><td>Session (β_2)</td><td>0.11688</td><td>1.7619</td><td>0.066338</td><td>0.94729</td></tr>
</table>

Number of observations: 76, Error degrees of freedom: 73
Root Mean Squared Error: 214
R-squared: 0.0353, Adjusted R-Squared 0.00891
F-statistic vs. constant model: 1.34, p-value = 0.269</td></tr>
</table>

<table>
<tr><th>Patient</th><th>Memory Paths</th><th>Regression results</th></tr>
<tr><td>5</td><td>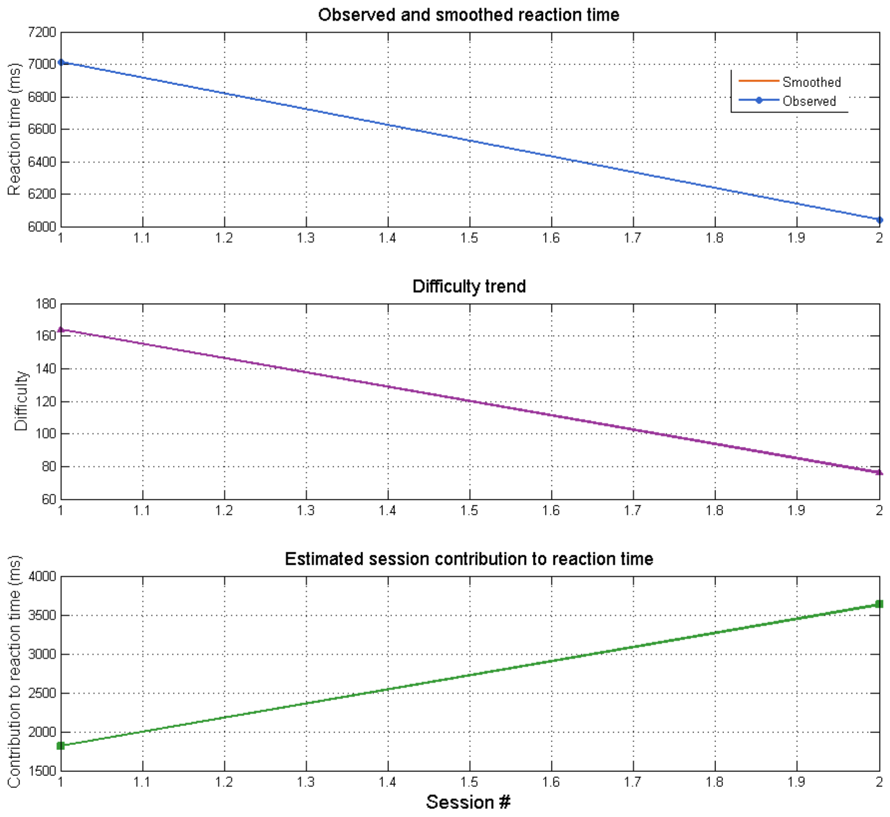
</td><td>Estimated Coefficients:
Estimate SE tStat pValue
________ __ _____ ______

(Intercept) 0 0 NaN NaN
Difficulty 31.688 0 Inf NaN
Session (β_2) 1817.2 0 Inf NaN

Number of observations: 2, Error degrees of freedom: 0
Root Mean Squared Error: 0
R-squared: 1, Adjusted R-Squared NaN
F-statistic vs. constant model: NaN, p-value = NaN</td></tr>
</table>

<table>
<tr><th>Patient</th><th>Memory Paths</th><th>Regression results</th></tr>
<tr><td>1</td><td>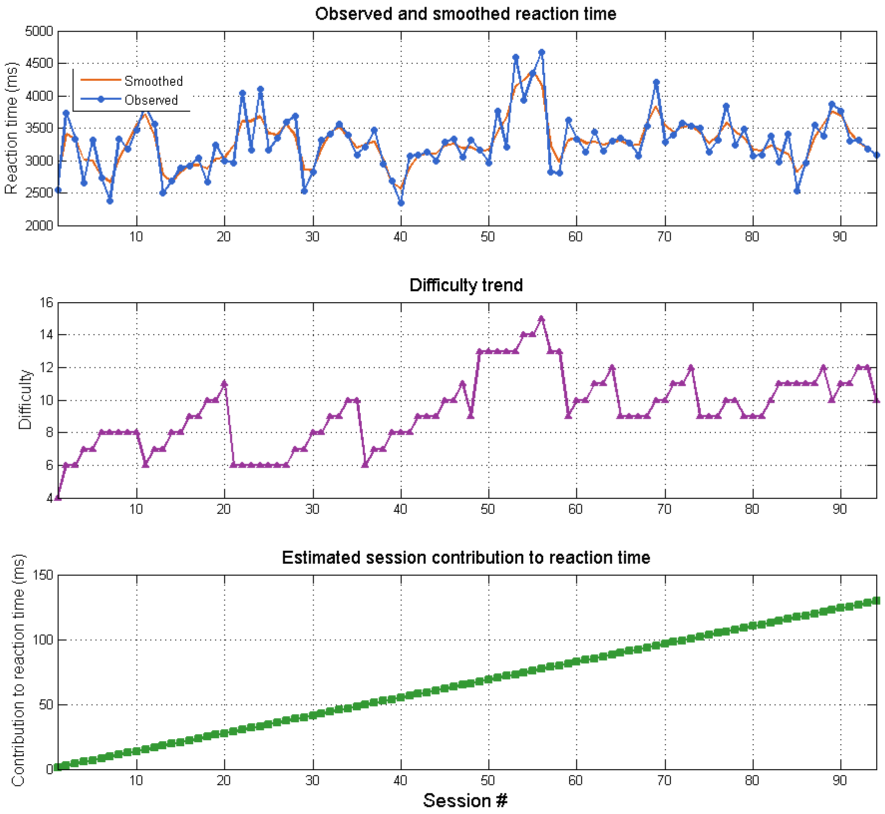
</td><td>Estimated Coefficients:

<table>
<tr><th></th><th>Estimate</th><th>SE</th><th>tStat</th><th>pValue</th></tr>
<tr><td>(Intercept)</td><td>2820.5</td><td>146.78</td><td>19.216</td><td>8.6535e-34</td></tr>
<tr><td>Difficulty</td><td>41.547</td><td>18.78</td><td>2.2124</td><td>0.029445</td></tr>
<tr><td>Session (β_2)</td><td>1.3768</td><td>1.5405</td><td>0.89375</td><td>0.37381</td></tr>
</table>

Number of observations: 94, Error degrees of freedom: 91
Root Mean Squared Error: 320
R-squared: 0.125, Adjusted R-Squared 0.106
F-statistic vs. constant model: 6.52, p-value = 0.00225</td></tr>
</table>

Patient	Memory Paths	Regression results
2	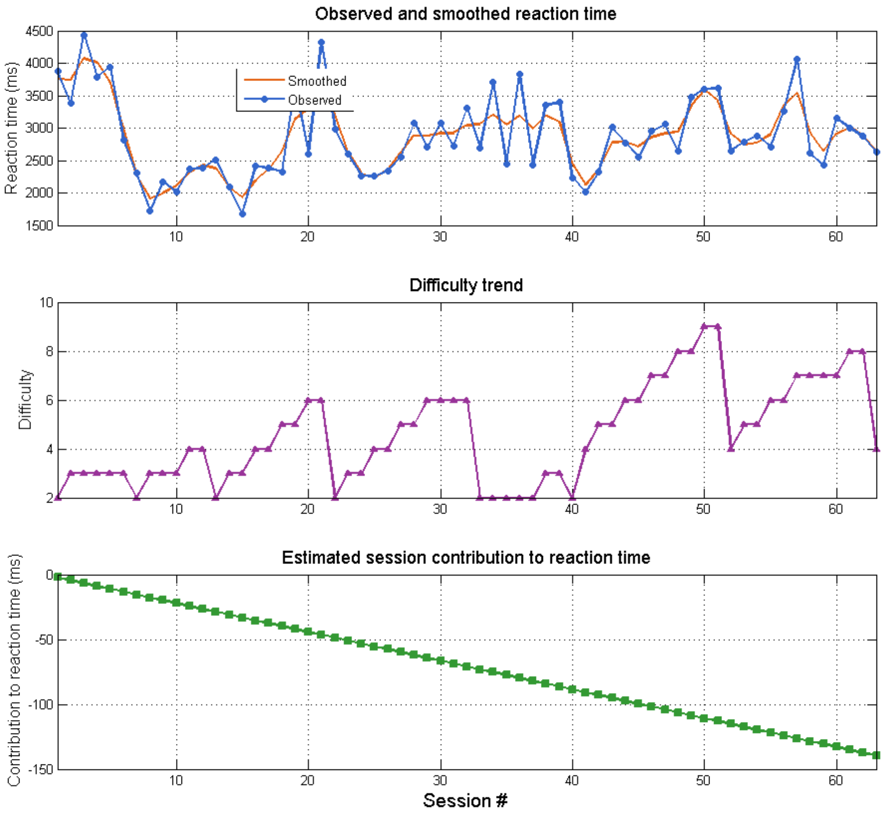 	Estimated Coefficients:<br>Estimate SE tStat pValue<br>________ ______ ________ __________<br>(Intercept) 2639.3 163.2 16.172 1.5197e-23<br>Difficulty 63.588 41.795 1.5214 0.1334<br>Session (β_2) -2.2157 4.5517 -0.48677 0.6282<br><br>Number of observations: 63, Error degrees of freedom: 60<br>Root Mean Squared Error: 507<br>R-squared: 0.0431, Adjusted R-Squared 0.0112<br>F-statistic vs. constant model: 1.35, p-value = 0.266

<table>
<tr><th>Patient</th><th>Memory Paths</th><th>Regression results</th></tr>
<tr><td>6</td><td>

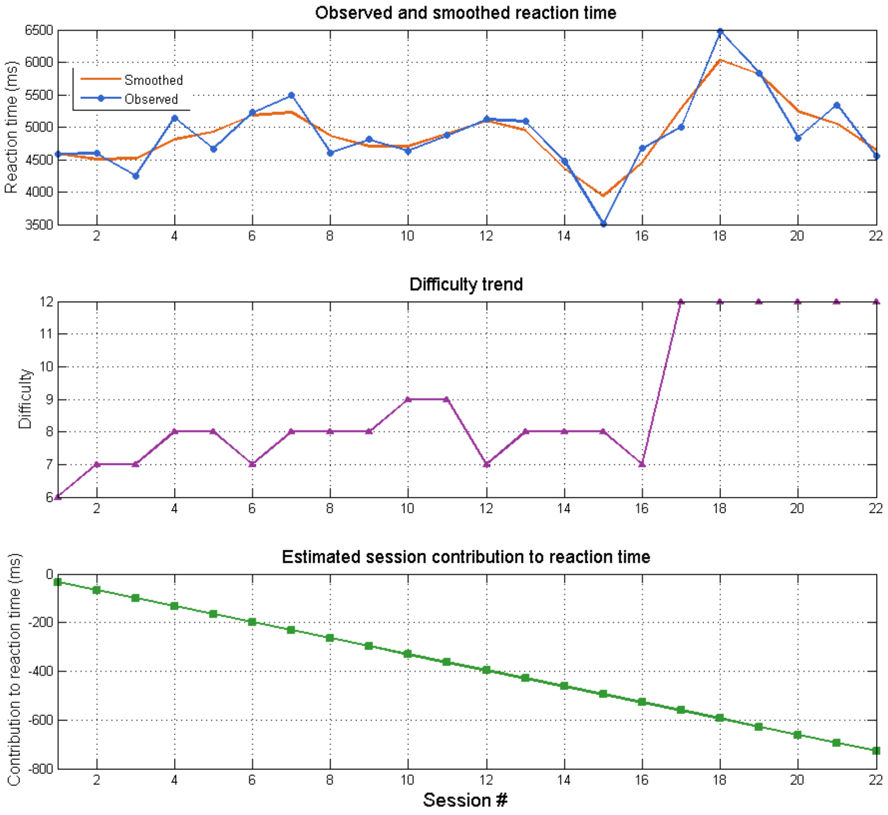

</td><td>

Estimated Coefficients:

	Estimate	SE	tStat	pValue
(Intercept)	3378.4	433.6	7.7914	2.4759e-07
Difficulty	214.46	67.99	3.1543	0.0052217
Session (β_2)	-33.04	21.747	-1.5193	0.14516

Number of observations: 22, Error degrees of freedom: 19
Root Mean Squared Error: 379
R-squared: 0.408, Adjusted R-Squared 0.345
F-statistic vs. constant model: 6.54, p-value = 0.0069

</td></tr>
</table>

<table>
<tr><th>Patient</th><th>Memory Paths</th><th>Regression results</th></tr>
<tr><td>4</td><td>

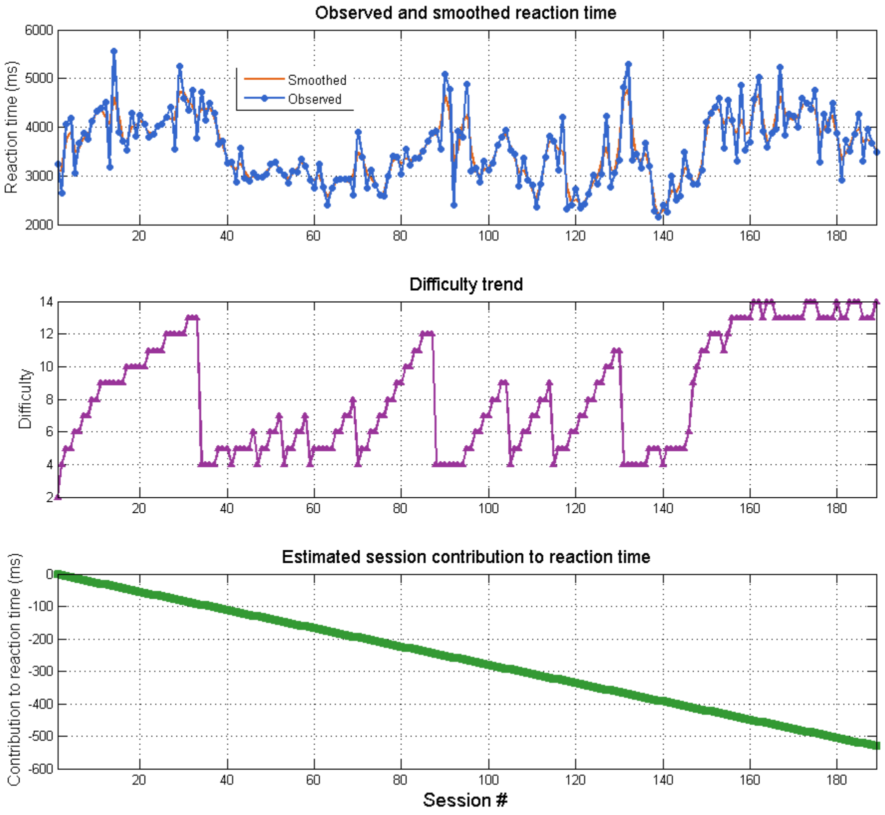

</td><td>

Estimated Coefficients:

	Estimate	SE	tStat	pValue
(Intercept)	2926.5	100.45	29.133	3.125e-71
Difficulty	107.39	11.975	8.9681	3.2175e-16
Session (β_2)	-2.8115	0.75616	-3.7182	0.00026549

Number of observations: 189, Error degrees of freedom: 186
Root Mean Squared Error: 510
R-squared: 0.302, Adjusted R-Squared 0.294
F-statistic vs. constant model: 40.2, p-value = 3.01e-15

</td></tr>
</table>

**Patient**	Tangram	**Regression results**
7	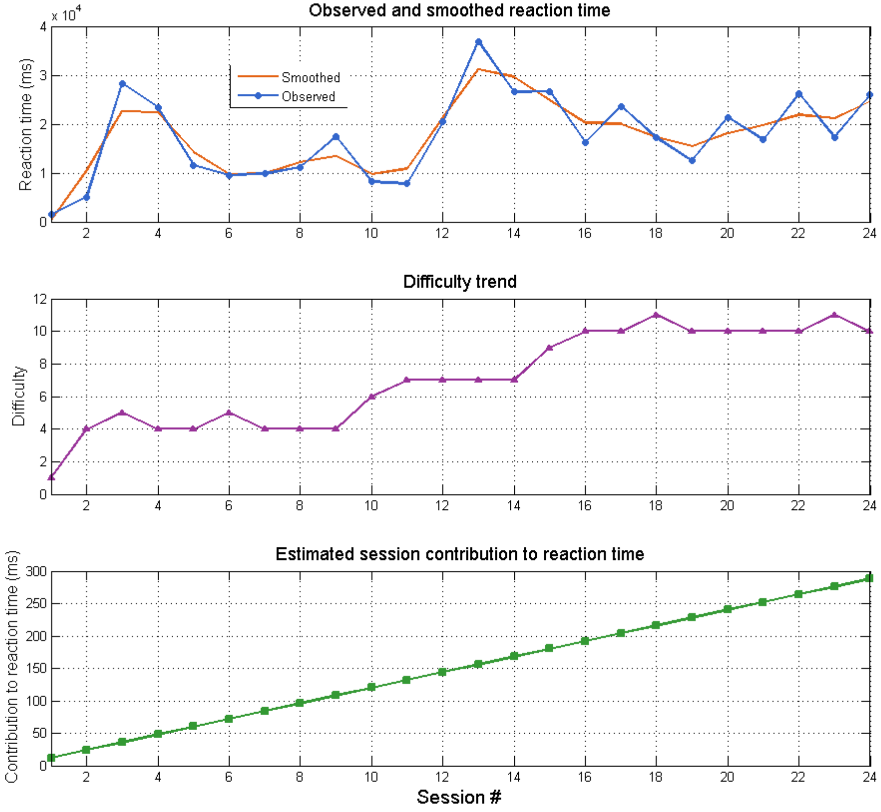	Estimated Coefficients:<br>Estimate SE tStat pValue<br>_________ _______ _________ _________<br>(Intercept) 8161.4 3900.1 2.0926 0.048709<br>Difficulty 1317.4 1284.1 1.026 0.31657<br>Session (β_2) 12.002 532.58 0.022535 0.98223<br><br>Number of observations: 24, Error degrees of freedom: 21<br>Root Mean Squared Error: 6.3e+03<br>R-squared: 0.3, Adjusted R-Squared 0.233<br>F-statistic vs. constant model: 4.5, p-value = 0.0236

<table>
<tr><th>Patient</th><th>Tangram</th><th>Regression results</th></tr>
<tr><td>5</td><td>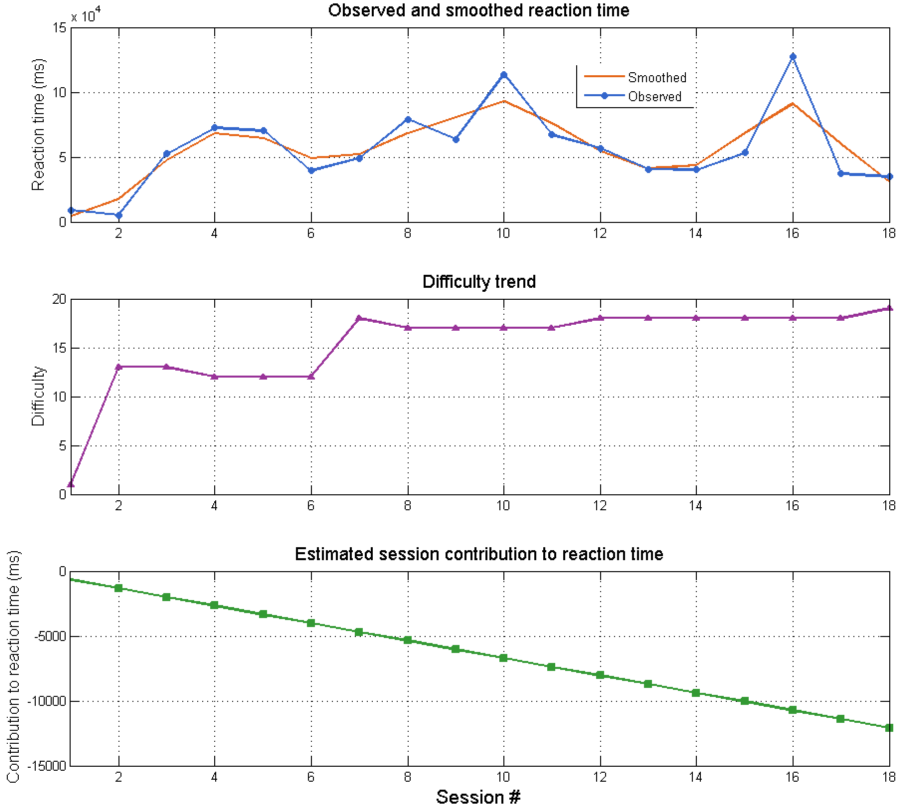
</td><td>Estimated Coefficients:

	Estimate	SE	tStat	pValue
(Intercept)	9282.7	20186	0.45986	0.65222
Difficulty	3472.9	1842.2	1.8852	0.078925
Session (β_2)	-671.48	1506.5	-0.44573	0.66216

Number of observations: 18, Error degrees of freedom: 15
Root Mean Squared Error: 2.13e+04
R-squared: 0.285, Adjusted R-Squared 0.19
F-statistic vs. constant model: 2.99, p-value = 0.0805</td></tr>
</table>

<table>
<tr><th>Patient</th><th>Tangram</th><th>Regression results</th></tr>
<tr><td>1</td><td>
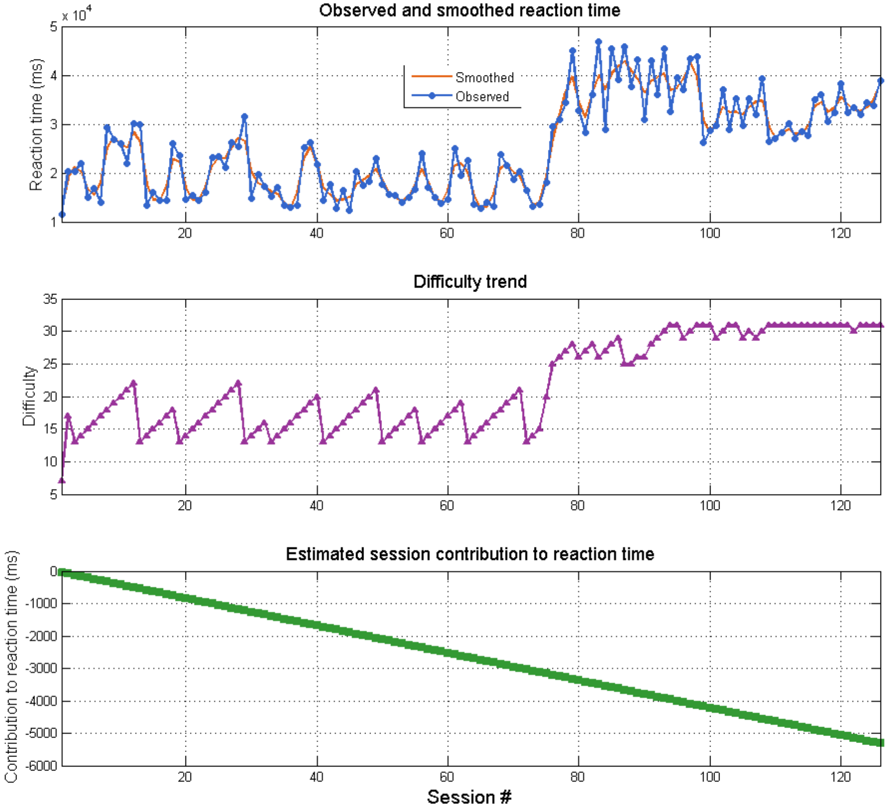

</td><td>
Estimated Coefficients:
Estimate SE tStat pValue

(Intercept) -532.27 1527.8 -0.3484 0.72814
Difficulty 1313.6 110.79 11.856 4.3056e-22
Session (β_2) -42.203 20.765 -2.0324 0.044267

Number of observations: 126, Error degrees of freedom: 123
Root Mean Squared Error: 4.58e+03
R-squared: 0.743, Adjusted R-Squared 0.739
F-statistic vs. constant model: 178, p-value = 4.8e-37
</td></tr>
</table>

**Patient**	Tangram	**Regression results**
2	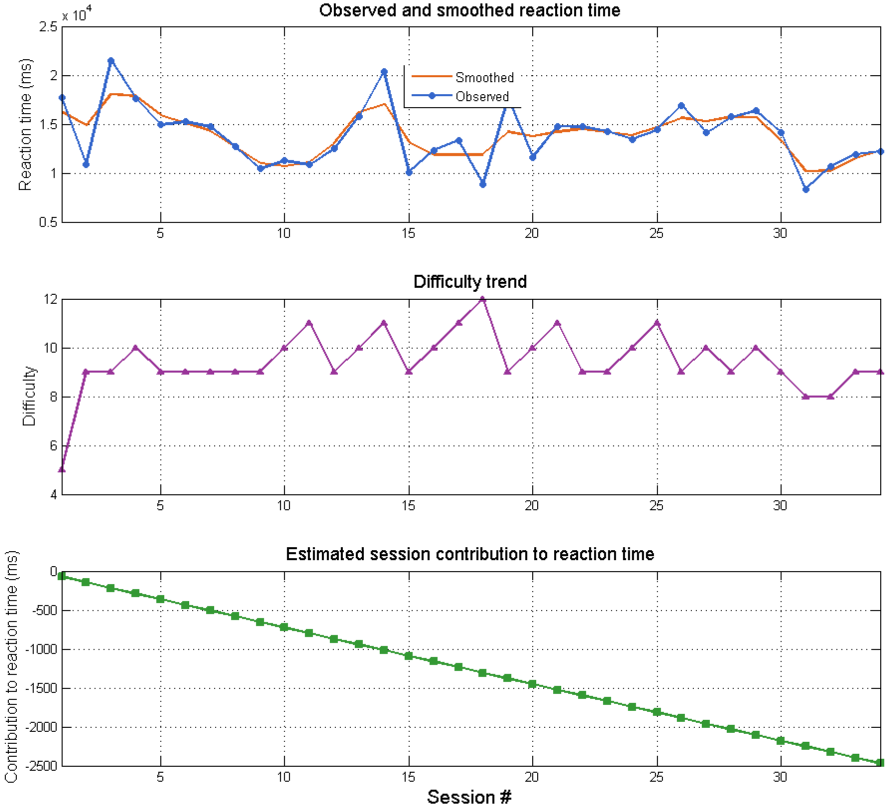	Estimated Coefficients:<br>Estimate SE tStat pValue<br>(Intercept) 15593 2870 5.4331 6.203e-06<br>Difficulty -41.276 301.39 -0.13695 0.89195<br>Session (β_2) -72.702 36.647 -1.9839 0.056188<br><br>Number of observations: 34, Error degrees of freedom: 31<br>Root Mean Squared Error: 2.08e+03<br>R-squared: 0.116, Adjusted R-Squared 0.0588<br>F-statistic vs. constant model: 2.03, p-value = 0.148

**Patient**	Tangram	**Regression results**
6	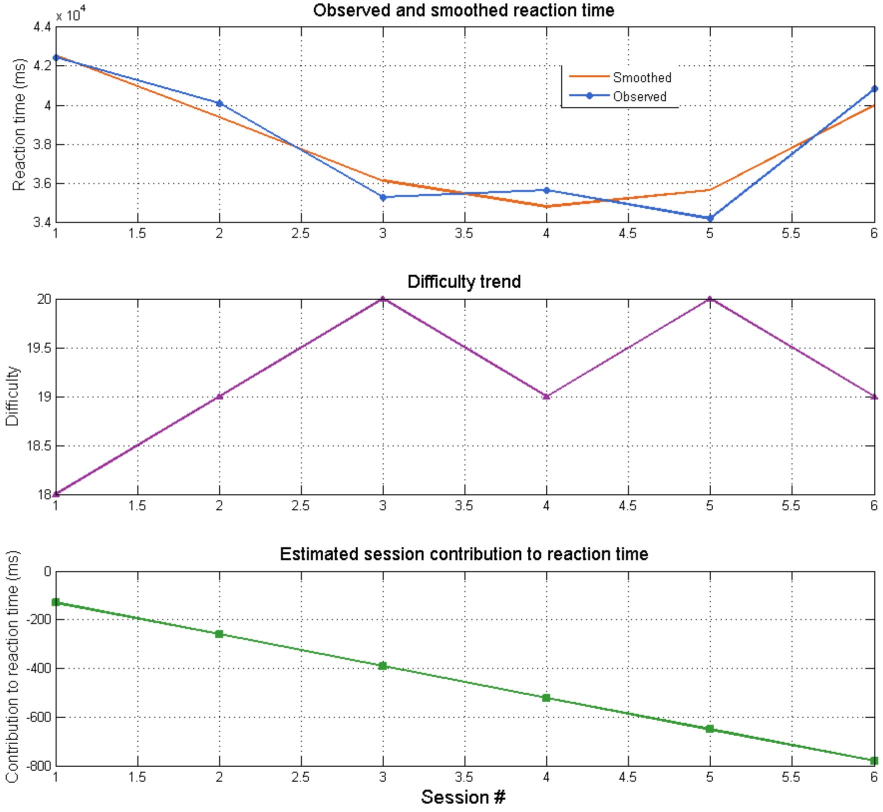	Estimated Coefficients:<br>Estimate SE tStat pValue<br>________ _____ ________ ________<br>(Intercept) 95851 31127 3.0794 0.054162<br>Difficulty -2990.7 1681 -1.7791 0.17328<br>Session (β_2) -130.26 676.4 -0.19257 0.85959<br><br>Number of observations: 6, Error degrees of freedom: 3<br>Root Mean Squared Error: 2.46e+03<br>R-squared: 0.611, Adjusted R-Squared 0.351<br>F-statistic vs. constant model: 2.35, p-value = 0.243

Patient	Tangram	Regression results
4	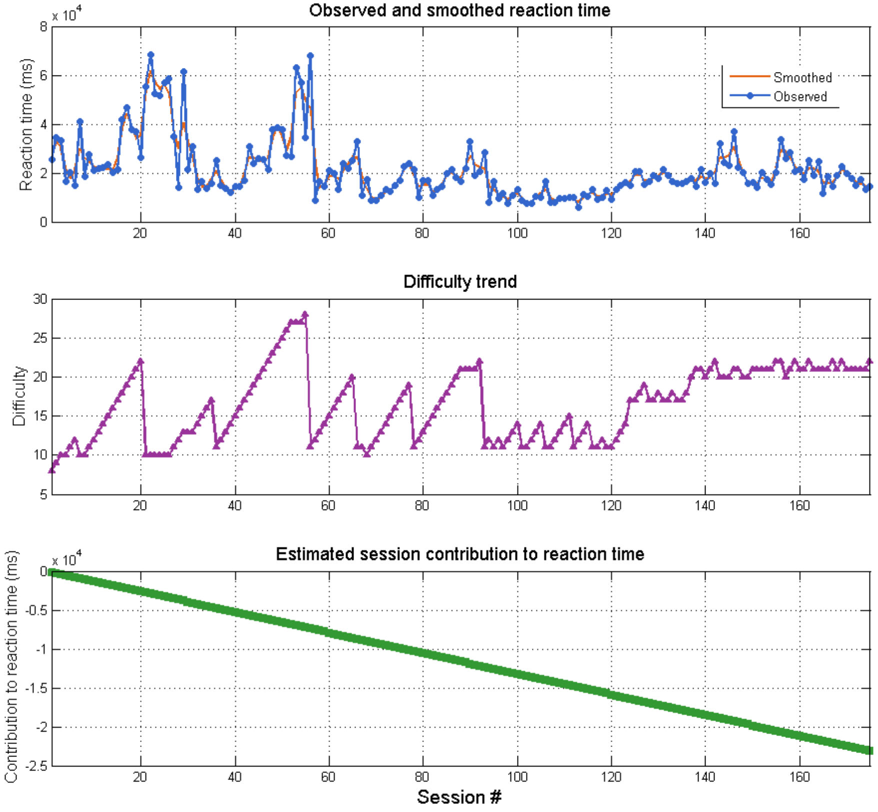 	Estimated Coefficients:<br>Estimate SE tStat pValue<br>________ ______ _______ __________<br><br>(Intercept) 16050 2516.7 6.3773 1.605e-09<br>Difficulty 1039.9 162.63 6.3942 1.4682e-09<br>Session (β_2) -132.49 14.659 -9.0384 3.2318e-16<br><br>Number of observations: 175, Error degrees of freedom: 172<br>Root Mean Squared Error: 8.88e+03<br>R-squared: 0.343, Adjusted R-Squared 0.335<br>F-statistic vs. constant model: 44.9, p-value = 2.08e-16
