# Supplementary material for: More than Just a Game: A Longitudinal Pilot Study on the Outcome Effects of Home-Based Digital Cognitive Rehabilitation in Outpatients with Mild Cognitive Impairment
Source: Brain Sci. 2026 May 29;16(6):582. doi: 10.3390/brainsci16060582 (PMC13297541; doi:10.3390/brainsci16060582)
Supplement: Supplementary file 1 [file brainsci-16-00582-s001.zip › brainsci-4309575-supplementary/Supplementary Materials/S3 - Radar Plots .pdf]

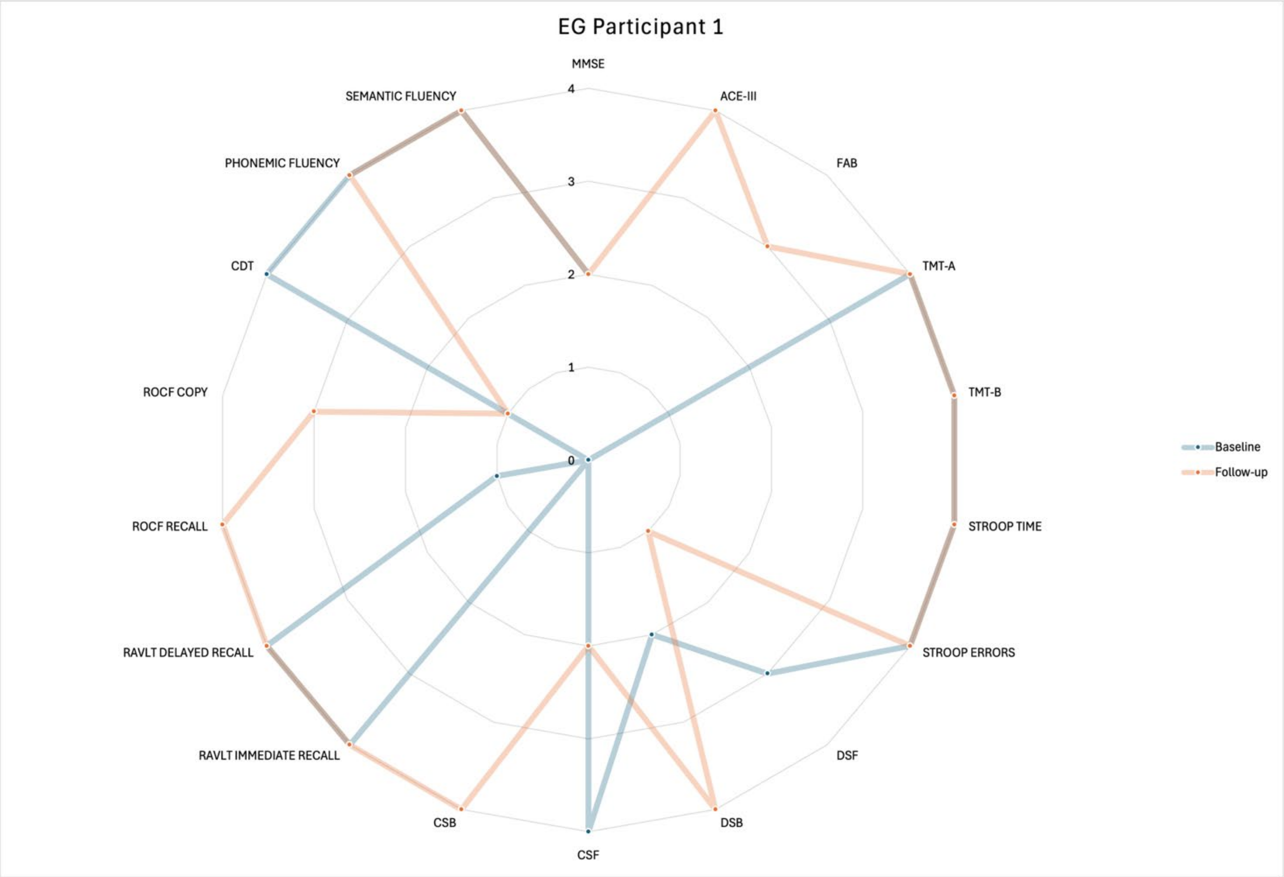
EG Participant 1
MMSE
ACE-III
FAB
TMT-A
TMT-B
STROOP TIME
STROOP ERRORS
DSF
DSB
CSF
CSB
RAVLT IMMEDIATE RECALL
RAVLT DELAYED RECALL
ROCF RECALL
ROCF COPY
CDT
PHONEMIC FLUENCY
SEMANTIC FLUENCY
0
1
2
3
4
Baseline
Follow-up

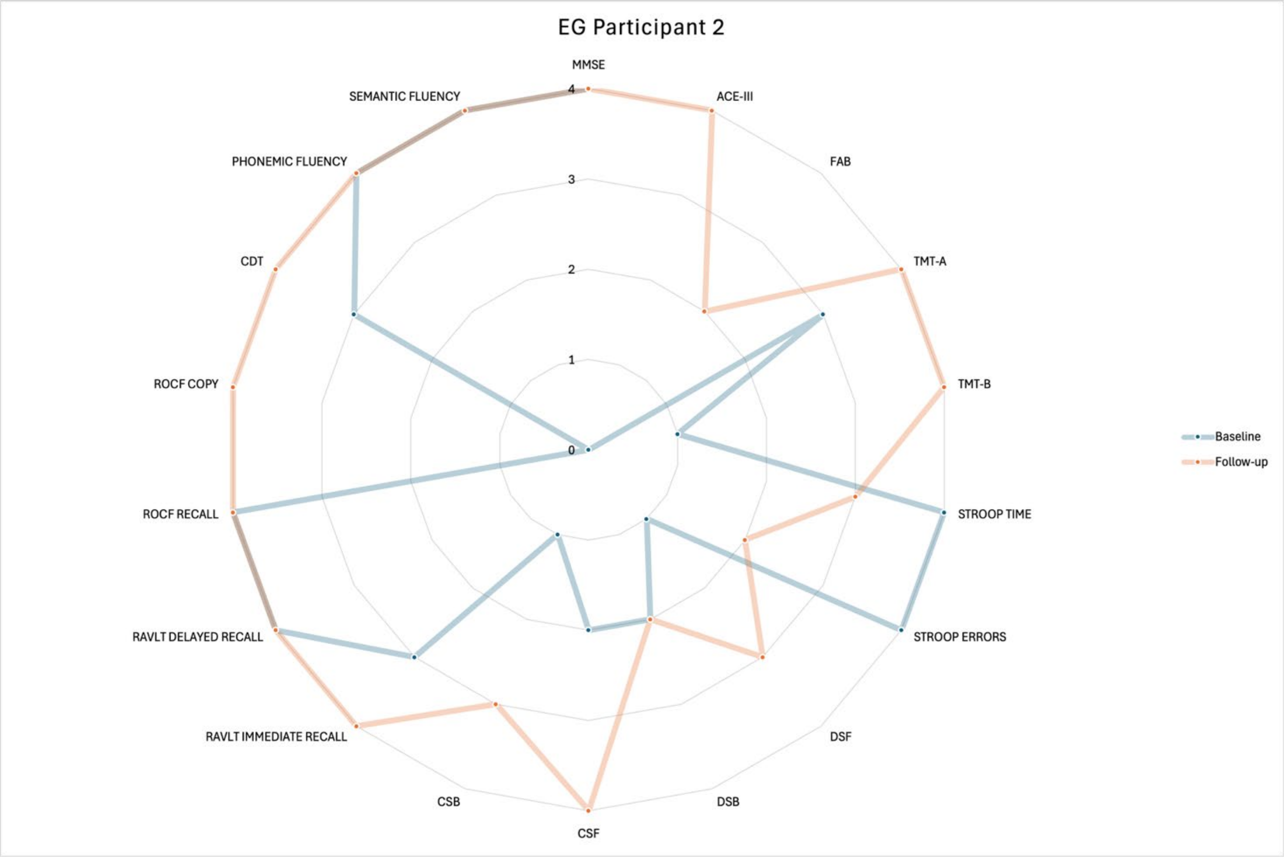
EG Participant 2
MMSE
ACE-III
FAB
TMT-A
TMT-B
STROOP TIME
STROOP ERRORS
DSF
DSB
CSF
CSB
RAVLT IMMEDIATE RECALL
RAVLT DELAYED RECALL
ROCF RECALL
ROCF COPY
CDT
PHONEMIC FLUENCY
SEMANTIC FLUENCY
0
1
2
3
4
Baseline
Follow-up

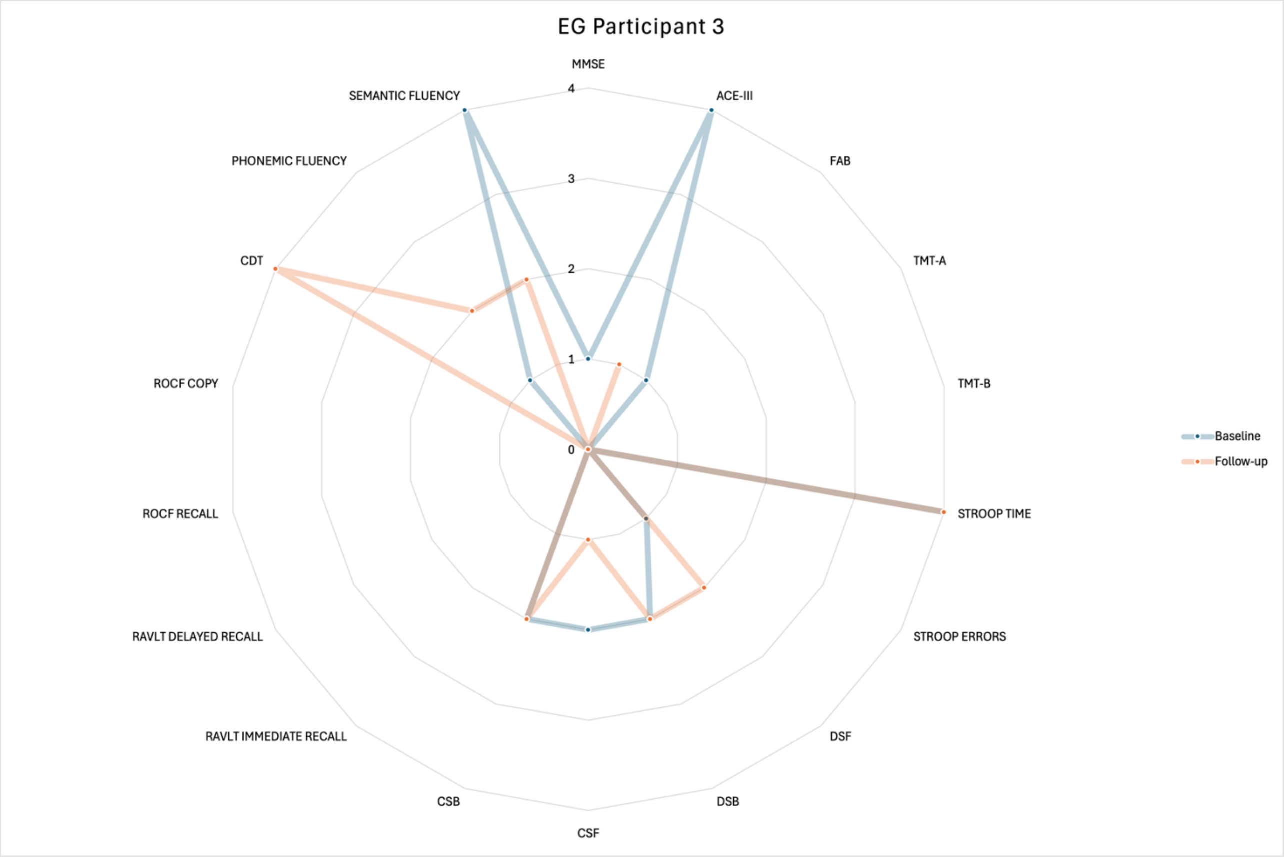
EG Participant 3
MMSE
ACE-III
FAB
TMT-A
TMT-B
STROOP TIME
STROOP ERRORS
DSF
DSB
CSF
CSB
RAVLT IMMEDIATE RECALL
RAVLT DELAYED RECALL
ROCF RECALL
ROCF COPY
CDT
PHONEMIC FLUENCY
SEMANTIC FLUENCY
0
1
2
3
4
Baseline
Follow-up

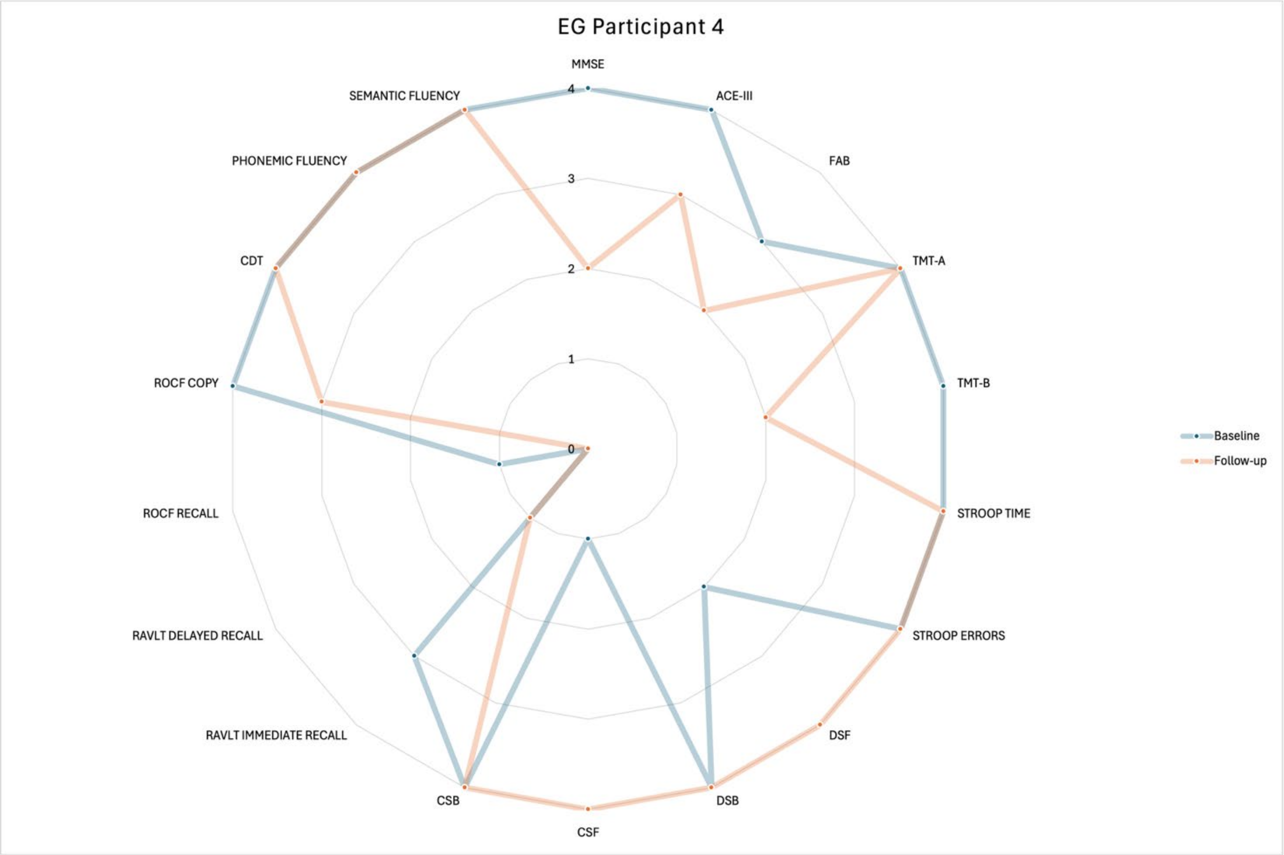
EG Participant 4
MMSE
ACE-III
FAB
TMT-A
TMT-B
STROOP TIME
STROOP ERRORS
DSF
DSB
CSF
CSB
RAVLT IMMEDIATE RECALL
RAVLT DELAYED RECALL
ROCF RECALL
ROCF COPY
CDT
PHONEMIC FLUENCY
SEMANTIC FLUENCY
0
1
2
3
4
Baseline
Follow-up

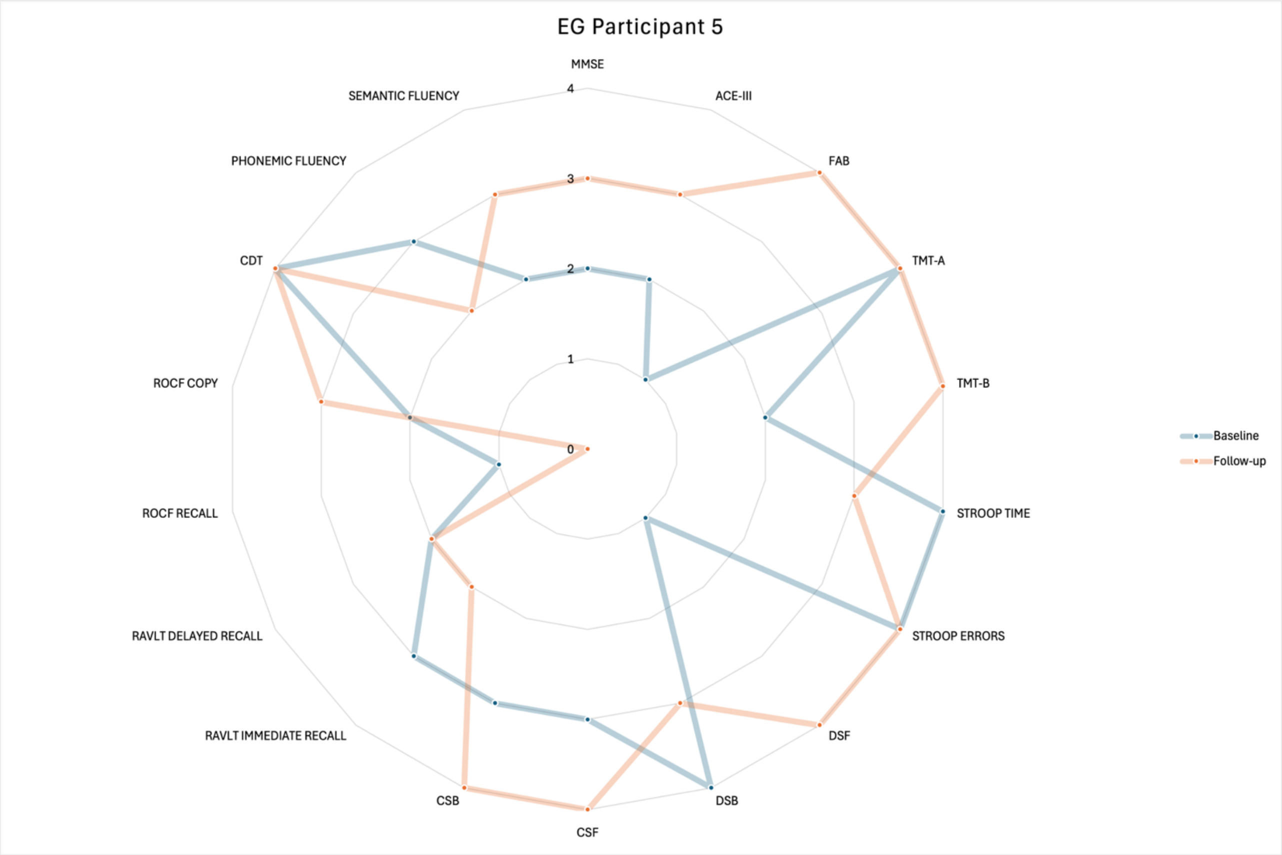
EG Participant 5
MMSE
ACE-III
FAB
TMT-A
TMT-B
STROOP TIME
STROOP ERRORS
DSF
DSB
CSF
CSB
RAVLT IMMEDIATE RECALL
RAVLT DELAYED RECALL
ROCF RECALL
ROCF COPY
CDT
PHONEMIC FLUENCY
SEMANTIC FLUENCY
0
1
2
3
4
Baseline
Follow-up

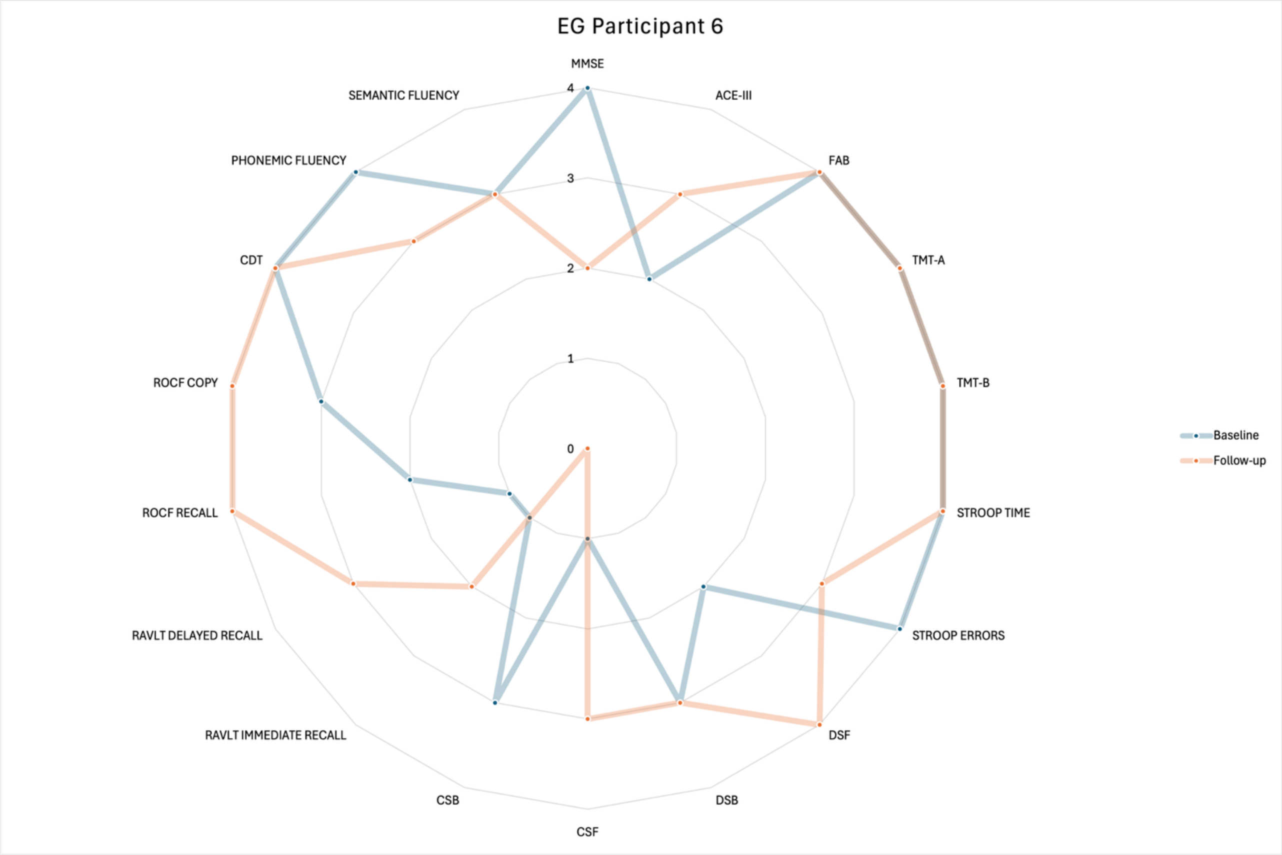
EG Participant 6
MMSE
ACE-III
FAB
TMT-A
TMT-B
STROOP TIME
STROOP ERRORS
DSF
DSB
CSF
CSB
RAVLT IMMEDIATE RECALL
RAVLT DELAYED RECALL
ROCF RECALL
ROCF COPY
CDT
PHONEMIC FLUENCY
SEMANTIC FLUENCY
0
1
2
3
4
Baseline
Follow-up

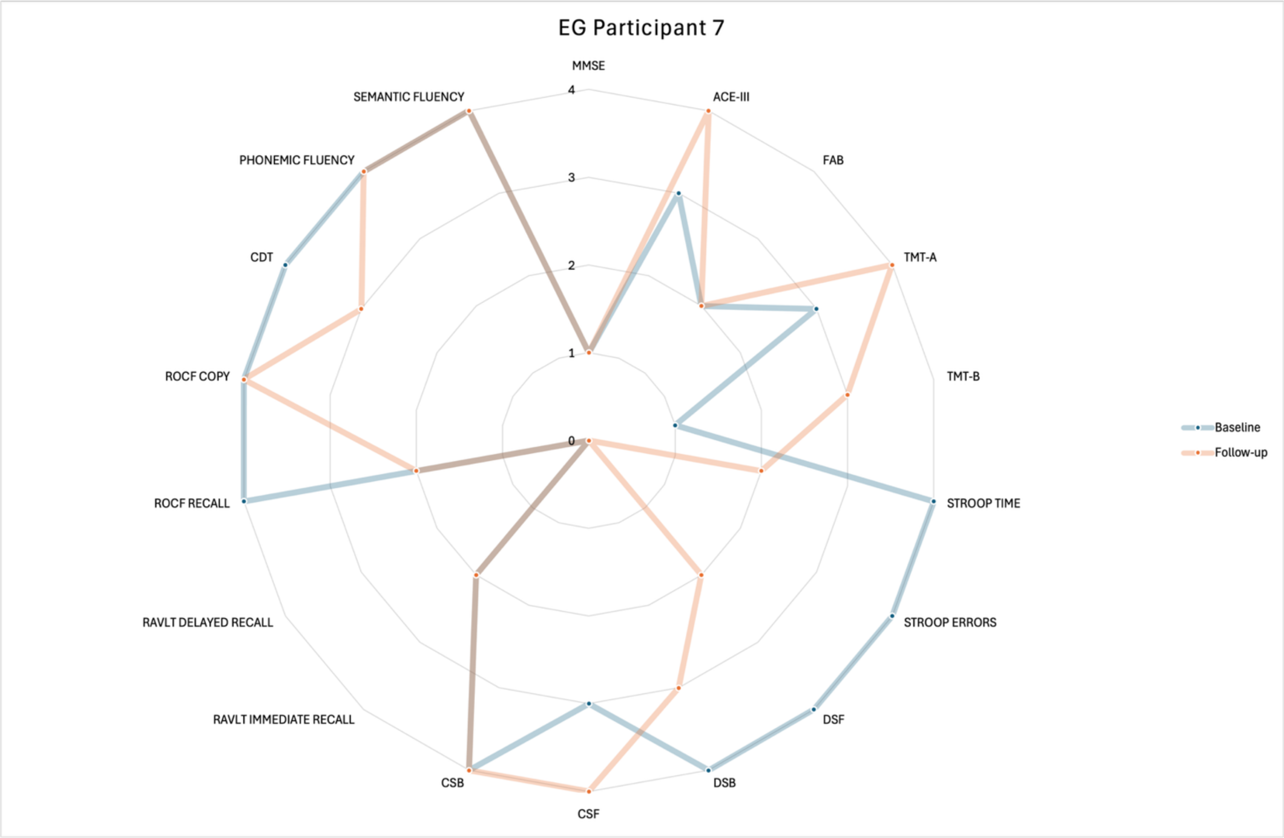

EG Participant 7
MMSE
ACE-III
FAB
TMT-A
TMT-B
STROOP TIME
STROOP ERRORS
DSF
DSB
CSF
CSB
RAVLT IMMEDIATE RECALL
RAVLT DELAYED RECALL
ROCF RECALL
ROCF COPY
CDT
PHONEMIC FLUENCY
SEMANTIC FLUENCY
4
3
2
1
0
Baseline
Follow-up

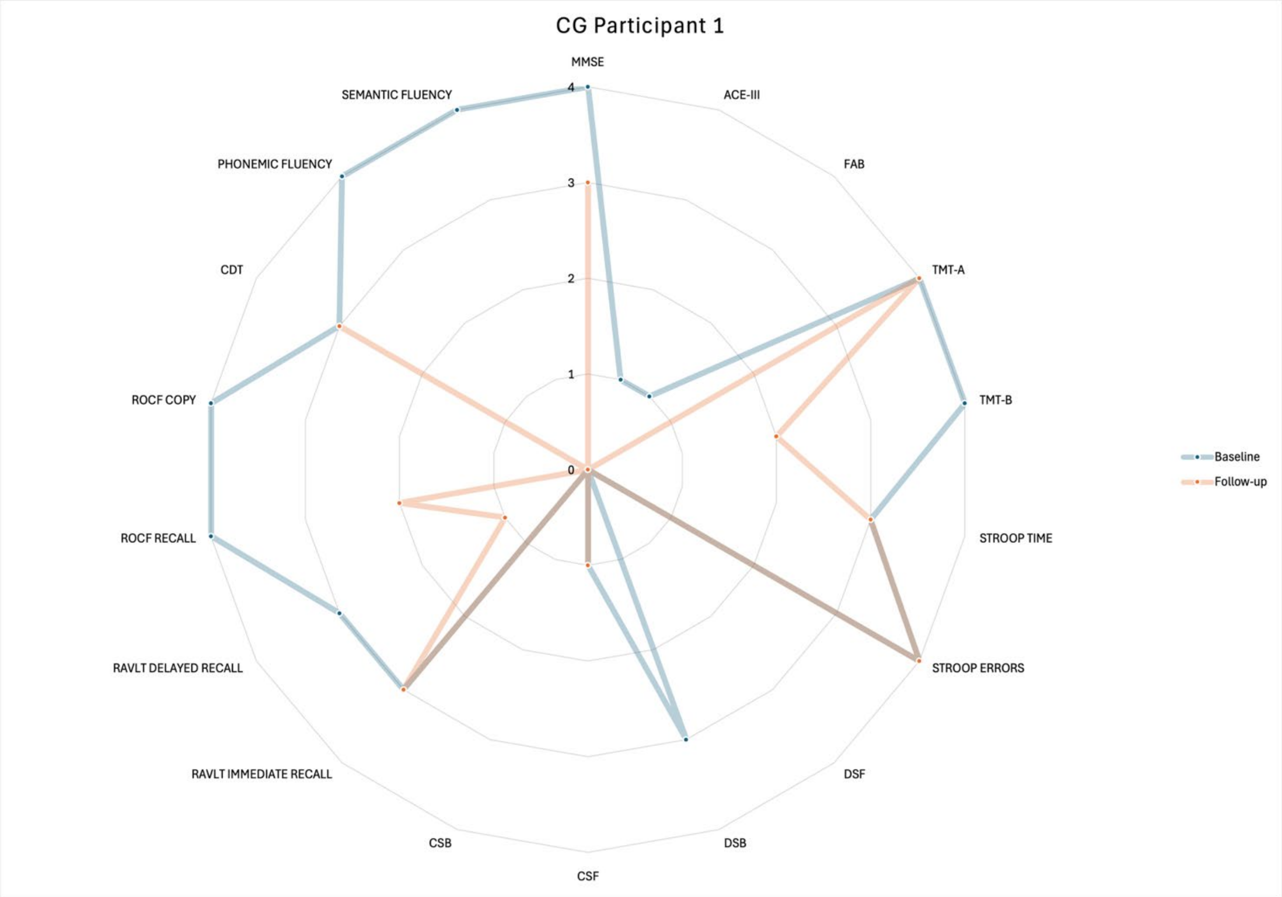
CG Participant 1
MMSE
ACE-III
FAB
TMT-A
TMT-B
STROOP TIME
STROOP ERRORS
DSF
DSB
CSF
CSB
RAVLT IMMEDIATE RECALL
RAVLT DELAYED RECALL
ROCF RECALL
ROCF COPY
CDT
PHONEMIC FLUENCY
SEMANTIC FLUENCY
0
1
2
3
4
Baseline
Follow-up

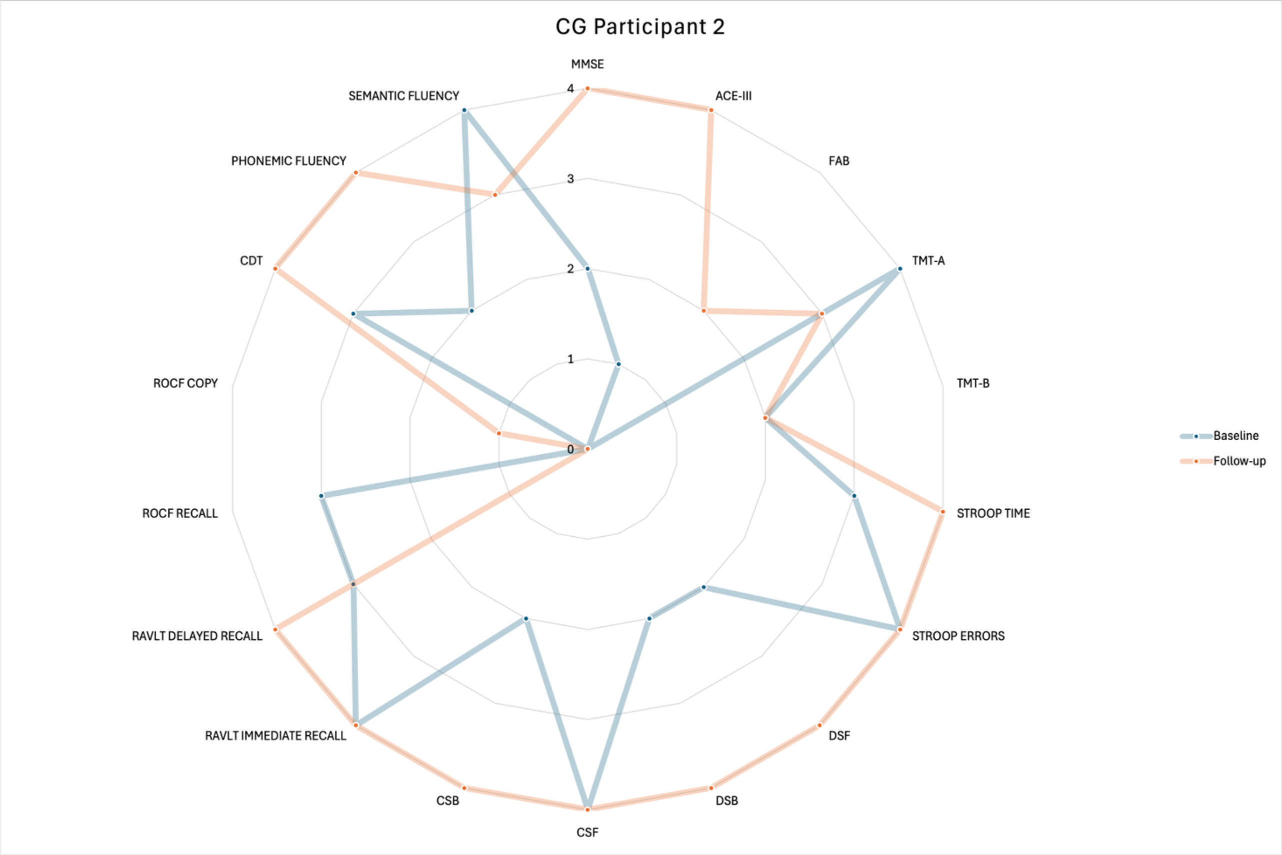
CG Participant 2
MMSE
ACE-III
FAB
TMT-A
TMT-B
STROOP TIME
STROOP ERRORS
DSF
DSB
CSF
CSB
RAVLT IMMEDIATE RECALL
RAVLT DELAYED RECALL
ROCF RECALL
ROCF COPY
CDT
PHONEMIC FLUENCY
SEMANTIC FLUENCY
0
1
2
3
4
Baseline
Follow-up

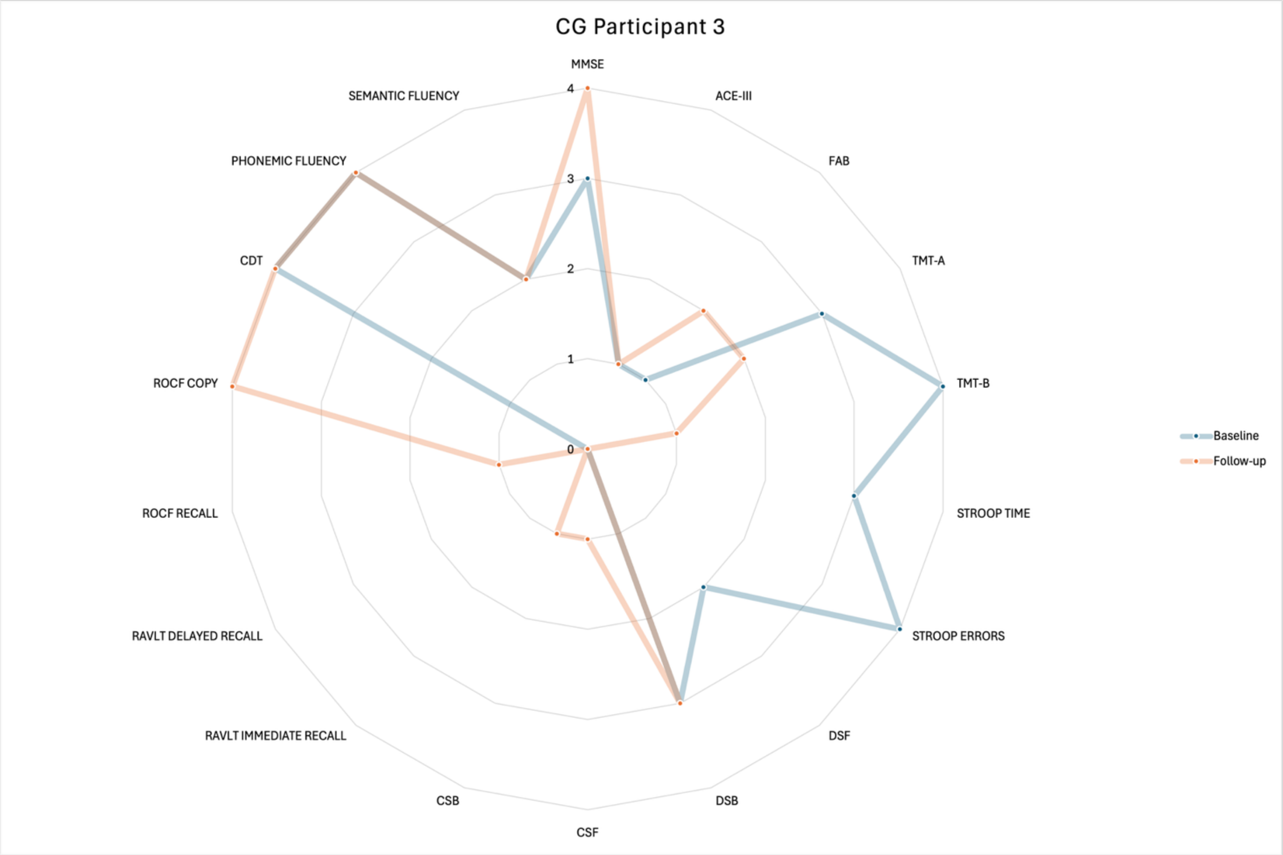
CG Participant 3
MMSE
ACE-III
FAB
TMT-A
TMT-B
STROOP TIME
STROOP ERRORS
DSF
DSB
CSF
CSB
RAVLT IMMEDIATE RECALL
RAVLT DELAYED RECALL
ROCF RECALL
ROCF COPY
CDT
PHONEMIC FLUENCY
SEMANTIC FLUENCY
0
1
2
3
4
Baseline
Follow-up

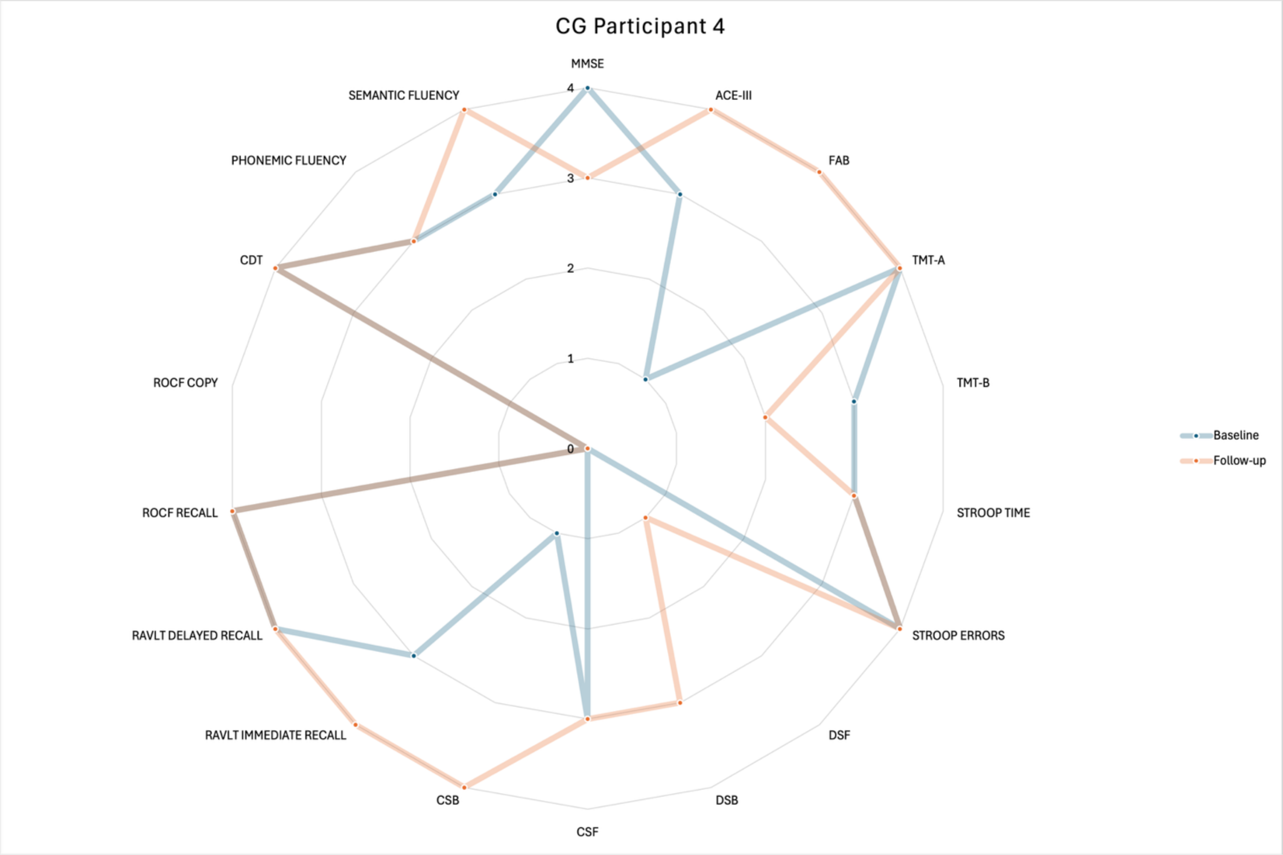
CG Participant 4
MMSE
ACE-III
FAB
TMT-A
TMT-B
STROOP TIME
STROOP ERRORS
DSF
DSB
CSF
CSB
RAVLT IMMEDIATE RECALL
RAVLT DELAYED RECALL
ROCF RECALL
ROCF COPY
CDT
PHONEMIC FLUENCY
SEMANTIC FLUENCY
0
1
2
3
4
Baseline
Follow-up

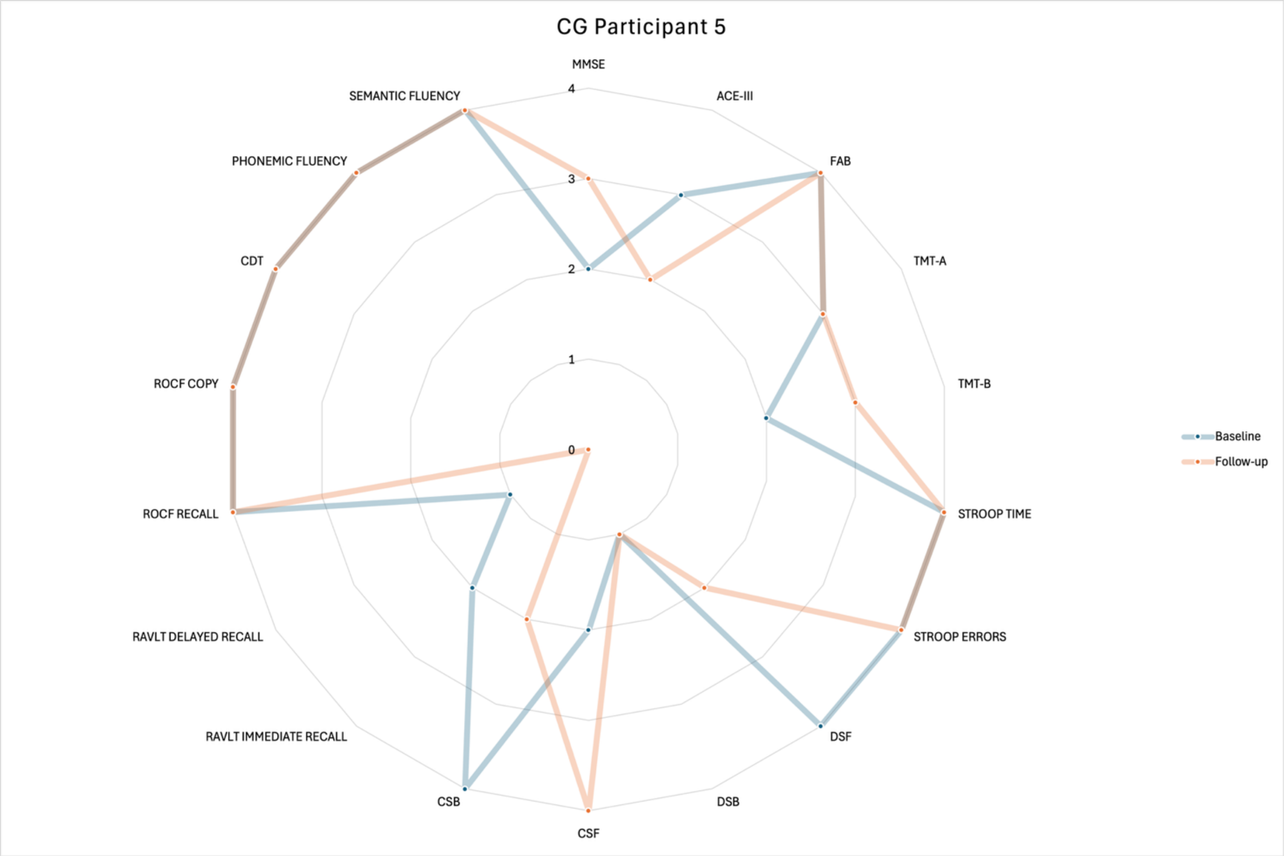
CG Participant 5
MMSE
ACE-III
FAB
TMT-A
TMT-B
STROOP TIME
STROOP ERRORS
DSF
DSB
CSF
CSB
RAVLT IMMEDIATE RECALL
RAVLT DELAYED RECALL
ROCF RECALL
ROCF COPY
CDT
PHONEMIC FLUENCY
SEMANTIC FLUENCY
0
1
2
3
4
Baseline
Follow-up

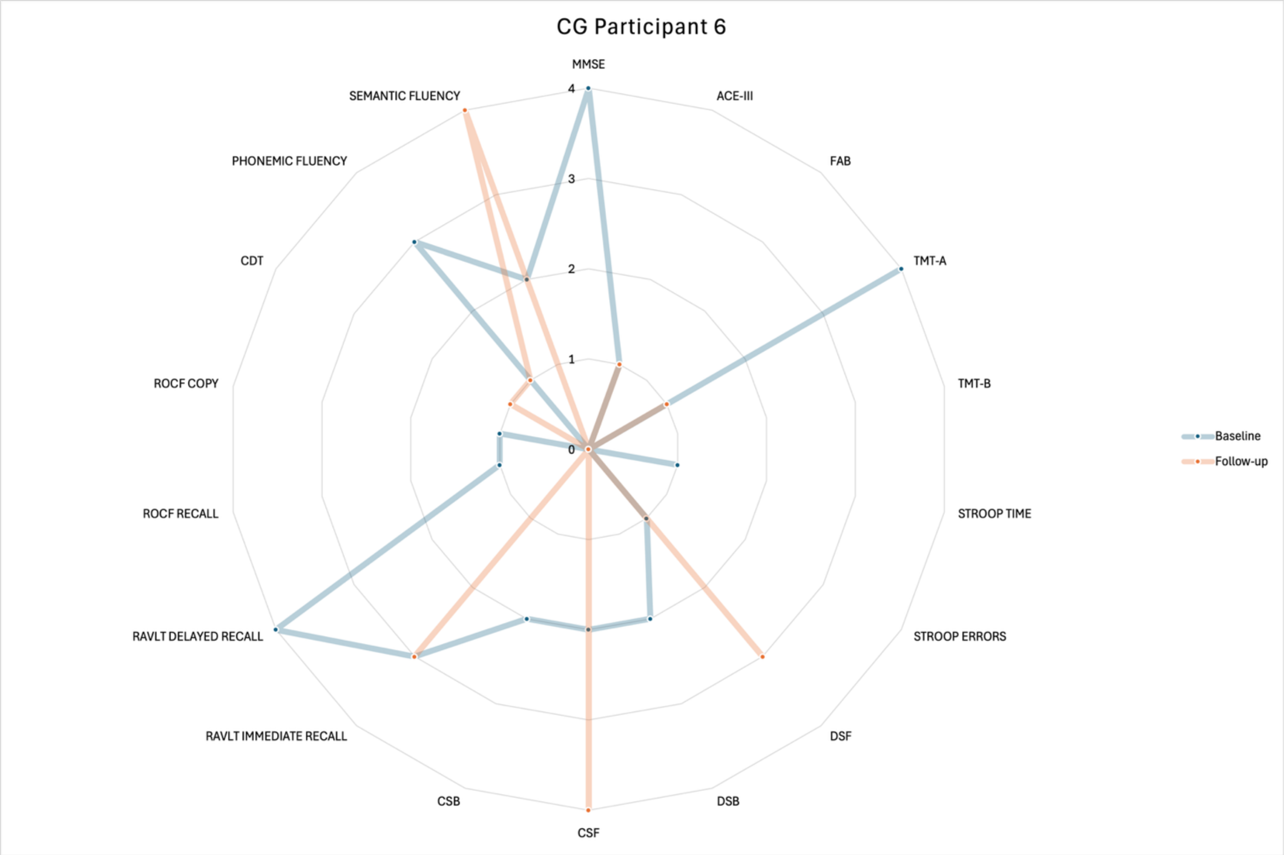
CG Participant 6
MMSE
ACE-III
FAB
TMT-A
TMT-B
STROOP TIME
STROOP ERRORS
DSF
DSB
CSF
CSB
RAVLT IMMEDIATE RECALL
RAVLT DELAYED RECALL
ROCF RECALL
ROCF COPY
CDT
PHONEMIC FLUENCY
SEMANTIC FLUENCY
0
1
2
3
4
Baseline
Follow-up

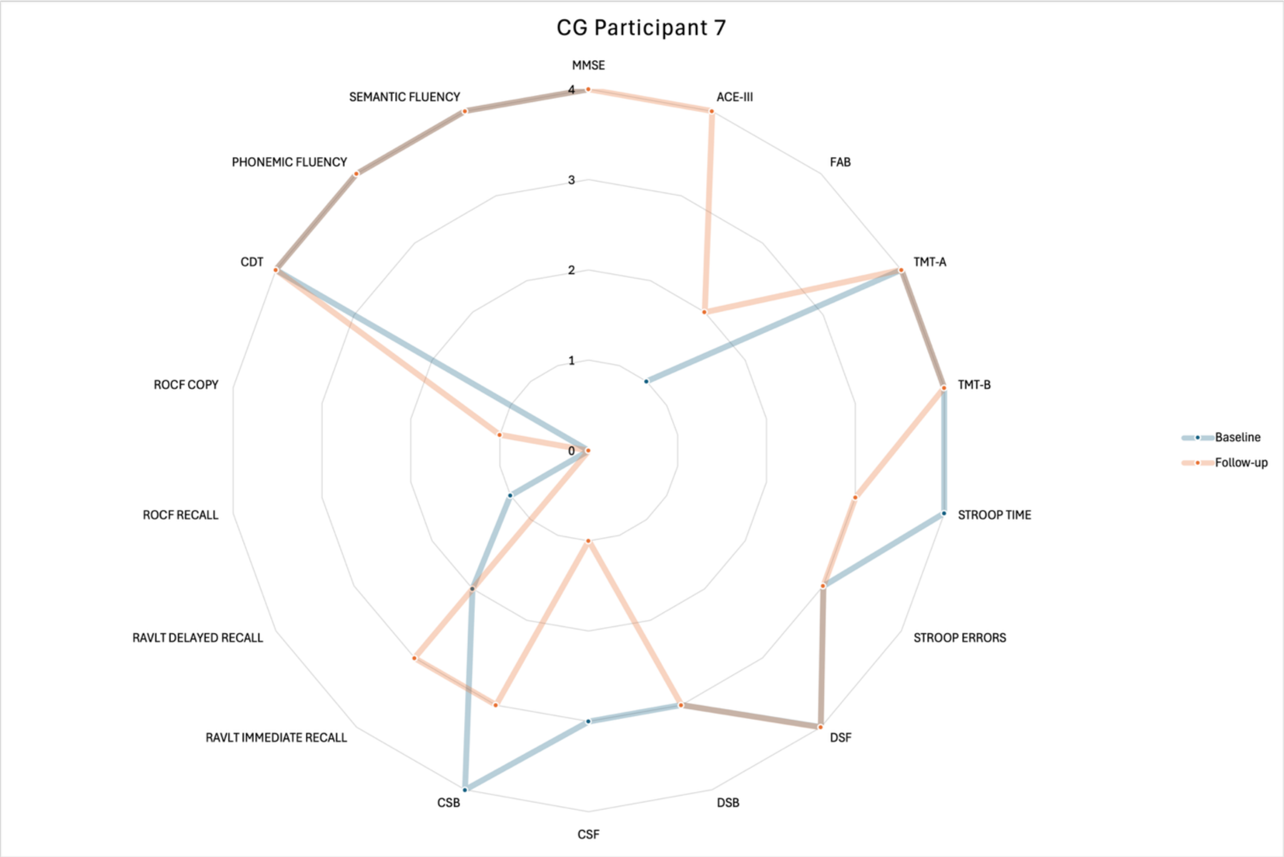
CG Participant 7
MMSE
ACE-III
FAB
TMT-A
TMT-B
STROOP TIME
STROOP ERRORS
DSF
DSB
CSF
CSB
RAVLT IMMEDIATE RECALL
RAVLT DELAYED RECALL
ROCF RECALL
ROCF COPY
CDT
PHONEMIC FLUENCY
SEMANTIC FLUENCY
0
1
2
3
4
Baseline
Follow-up
